# Pyroptosis and ferroptosis induced by mixed lineage kinase 3 (MLK3) signaling in cardiomyocytes are essential for myocardial fibrosis in response to pressure overload

**DOI:** 10.1038/s41419-020-02777-3

**Published:** 2020-07-24

**Authors:** Junyan Wang, Bo Deng, Qing Liu, Yusheng Huang, Weitao Chen, Jing Li, Zheng Zhou, Lu Zhang, Birong Liang, Jiaqi He, Zixin Chen, Cui Yan, Zhongqi Yang, Shaoxiang Xian, Lingjun Wang

**Affiliations:** 1 The First Affiliated Hospital, Guangzhou University of Chinese Medicine, Guangzhou, 510405 China; 2 The First Clinical Medical School, Guangzhou University of Chinese Medicine, Guangzhou, 510405 China; 3 Lingnan Medical Research Center, Guangzhou University of Chinese Medicine, Guangzhou, 510405 China; 4 Guangzhou Key Laboratory of Chinese Medicine for Prevention and Treatment of Chronic Heart Failure, Guangzhou, 510405 China; 5 National Clinical Research Base of Traditional Chinese Medicine, Guangzhou, 510405 China

**Keywords:** Molecular biology, Inflammasome

## Abstract

Chronic heart failure (CHF) is the final outcome of many cardiovascular diseases, and is a severe health issue faced by the elderly population. Mixed lineage kinase 3 (MLK3), a member of MAP3K family, is associated with aging, inflammation, oxidative stress, and related diseases, such as CHF. MLK3 has also been reported to play an important role in protecting against cardiomyocyte injury; however, its function in myocardial fibrosis is unknown. To investigate the role of MLK3 in myocardial fibrosis, we inhibited the expression of MLK3, and examined cardiac function and remodeling in TAC mice. In addition, we assessed the expression of MLK3 protein in ventricular cells and its downstream associated protein. We found that MLK3 mainly regulates NF-κB/NLRP3 signaling pathway-mediated inflammation and that pyroptosis causes myocardial fibrosis in the early stages of CHF. Similarly, MLK3 mainly regulates the JNK/p53 signaling pathway-mediated oxidative stress and that ferroptosis causes myocardial fibrosis in the advanced stages of CHF. We also found that promoting the expression of miR-351 can inhibit the expression of MLK3, and significantly improve cardiac function in mice subjected to TAC. These results suggest the pyroptosis and ferroptosis induced by MLK3 signaling in cardiomyocytes are essential for adverse myocardial fibrosis, in response to pressure overload. Furthermore, miR-351, which has a protective effect on ventricular remodeling in heart failure caused by pressure overload, may be a key target for the regulation of MLK3.

## Introduction

Chronic heart failure (CHF) characterized by high mortality and morbidity is the final outcome of many cardiovascular diseases^1,2^. Myocardial fibrosis is the major pathological process associated with myocardial remodeling. Almost every heart disease, like hypertension, cardiac valve disease, and myocardial ischemia, causes myocardial fibrosis, eventually leading to impaired systolic and diastolic function^3,4^. Myocardial fibrosis is a complex process, characterized by the accumulation of extracellular matrix proteins, which results in the expansion of the cardiac interstitium and the accumulation of scar tissue^5,6^. In recent years, inflammation and oxidative stress have been shown to play important roles in myocardial remodeling in CHF^7^.

Inflammation during the process of myocardial damage and repair leads to collagen deposits to form a collagen-based scar^8,9^. There is substantial evidence that CHF is associated with inflammation, regardless of whether it is caused by ischemic or nonischemic damage^10^. Nod-like receptor protein 3 (NLRP3) form a protein complex including caspase-1 and ASC called the NLRP3 inflammasome. The NLRP3 inflammasome can produce interleukin 1β (IL-1β) and IL-18^11^ and mediate pyroptosis, which is caspase-1-dependent programmed cell death, and is also known as inflammatory necrosis^12^. Recent research has shown that NLRP3 plays an important role in cardiovascular disease. Sano et al.^13–15^ found that NLRP3 inflammasome-targeted therapies might be effective methods to reduce infarct size and prevent heart failure following AMI and transverse aortic constriction (TAC).

Ferroptosis is a relatively newly discovered form of regulated cell death^16^. Accumulating evidence indicates that ferroptosis plays an important role in renal failure^17^, cardiovascular disease^18^, and other diseases^19,20^. Ferroptosis is iron-dependent cell death characterized by intracellular ROS accumulation^21^. ROS is known to play an important role in cardiovascular disease^22,23^, myocardial remodeling, contractile dysfunction, and structural alterations^24^. Glutathione (GSH), GSH peroxidase 4 (GPX4), heat shock protein beta-1, and nuclear factor erythroid 2-related factor 2 function as negative regulators of ferroptosis by limiting ROS production and reducing cellular iron uptake. In contrast, NADPH oxidase and p53 act as positive regulators of ferroptosis by promotion of ROS production and inhibition of expression of SLC7A11, respectively^21,25^. Previous studies indicate that ferroptosis might be another reason for the loss of cardiomyocytes in HF induced by pressure overload^26,27^.

Mixed lineage kinase 3 (MLK3), a member of MAP3K family and also known as MAP3K11, is thought to be involved in several diseases, including cancer, pulmonary fibrosis, and ischemic brain injury^28,29^. MLK3 is also thought to play an important role in protecting against cardiomyocyte injury, He et al.^30^ found that downregulation of MLK3 protects H9c2 cells from apoptosis and H/R injury induced by hypoxia. In addition, Zhang et al.^31^ has found that upregulation of miR-138 can protect H9c2 cells from hypoxia-induced cell death by negatively regulating MLK3. It also has been shown that MLK3 is closely related to inflammation and ROS^32–34^. p38 MAP kinase and c-Jun N-terminal kinase (JNK) are important downstream mediators of MLK3 signaling. Signaling through p38 MAPK can activate NF-κB expression to induce inflammation and JNK can activate p53 expression to induce ROS accumulation^35,36^. By sequencing and preliminary experiments, we found that MLK3 is highly expressed in TAC mice and closely related to inflammation and oxidative stress. Therefore, we observed the expression of MLK3 in the hearts of TAC mice at different periods, and determined whether MLK3 is involved in inflammation-mediated pyroptosis and ROS-mediated ferroptosis.

## Materials and methods

### Animal

Male wild-type (WT) C57BL/6J mice aged 8 weeks were obtained from the Experimental Animal Center, Guangzhou University of Chinese Medicine. C57BL/6J male mice were randomly assigned to eight groups: Sham, TAC, TAC + U-099, Sham + AAV^NC^, TAC + AAV^NC^, TAC + AAV^MLK3−^, TAC + atagomir, and TAC + agomir. TAC + U-099 mice were generated by intraperitoneal (i.p.) injection of URMC-099, an inhibitor of MLK3, has been reported to play an important role in a variety of diseases including anti-inflammatory and cognitive decline^34,37^ and as a useful tool to investigate the role of MLK3 in other diseases is common^38–40^ (10 mg/kg, dissolved in 10% DMSO, 40% PEG300, and 50% saline, every 12 h, MedChemExpress, Shanghai, China) 7 days before TAC surgery. Sham and TAC mice received corresponding isotype i.p. injections. TAC + AAV^MLK3−^ mice were generated by intravenous (i.v.) injection of adeno-associated viral vector-MLK3 vector (AAV^MLK3−^) (GenePharma, Shanghai, China) 14 and 21 days before TAC surgery. Sham + AAV^NC^ and TAC + AAV^NC^ mice received AAV^NC^ i.v. injections. TAC + antagomir and TAC + agomir were generated by i.v. injection of antagomir and agomir (30 pmol/g) 14 and 21 days before TAC surgery, respectively.

All animal studies were carried out with the approval of the Guangzhou University of Chinese Medicine Institutional Animal Care following the ethical code of animal use.

### Models and treatment

TAC surgery was used to establish CHF^41^. Briefly, mice were anesthetized by i.p. injection of pentobarbital sodium (50 mg/kg, sigma), and a thoracotomy was performed while mice were connected to a ventilator. The aorta was ligated between the right innominate artery and the left common carotid artery using an 8-0 silk suture ligature against a 27 G needle to yield a narrowing to 25–30% of its original cross-sectional area when the needle was removed. Sham-operated mice underwent the same thoracotomy procedure without the constriction of the aorta.

### Ultrasound echocardiography

Ultrasound echocardiography was performed by using a Vevo 2100 Imaging System (VisualSonics Inc., Toronto, ON, Canada) in mice under anesthesia with isoflurane (RWD Life Science Co., Guangdong, China). Briefly, mice were anesthetized with 1.0–2.5% isoflurane and heart rate were maintained ~450 and 550 beats/min. The heart was examined in the short-axis view at the papillary muscle level and an M-mode echocardiogram of the mid ventricle was recorded. Analysis of echocardiographic images was performed in a blinded manner. Cardiac function indices including left ventricular ejection fraction (LVEF), left ventricular fractional shortening (LVFS), end-diastolic left ventricular internal dimension (LVID; d), end-systolic left ventricular internal dimension (LVID; s), left ventricular end-diastolic volume (LVEDV), left ventricular end-systolic volume (LVESV), and left ventricular mass (LV mass).

### Histological examination

Hearts tissue was isolated and rinsed with phosphate buffered saline (PBS) and fixed in 4% paraformaldehyde (PFA) over 24 h. Then, the hearts were dehydrated and paraffin-embedded. Next, 5-µm-thick slices were cut for hematoxylin–eosin (H&E) staining to explore changes in heart size and Masson’s trichrome staining to visualize fibrosis. After staining all slices were completely scanned using Caseviewer 2.0 (Panoramic 250/MIDI, 3DHISTECH, Hungary). IPP 6.0 was used for morphometric analysis.

### Western blotting analysis

LV tissue was isolated and rinsed with PBS, then lysed using a Minute^TM^ Total Protein Extraction Kit for Animal Cultured Cells and Tissues (Ca. SD-001/SN-002, Invent Biotechnologies, USA). Protein content was measured via BCA Kit (Ca. P0012, Beyotime, Shanghai, China). Samples were heated at 95 °C with 2× loading buffer (Ca.FD003, Hangzhou Fude Biological Technology Co., Ltd, Hangzhou, China) for 5 min to fully denature proteins. Next, lysates were subjected to SDS-PAGE gel electrophoresis, and transferred to 0.45 µm PVDF membranes (Ca. 1620260, Bio-Rad Laboratories, Inc., USA). Membranes were the blocked with 5% skim milk (Ca. 9999, CST) at room temperature for 1 h and incubated overnight at 4 °C with 1:1000 GAPDH (CST, 2118), 1:1000 MLK3 (Proteintech, 11996-1-AP), 1:1000 Phospho-MLK3 (Thr277/Ser281, Abcam, ab191530), 1:1000 NF-κB p65 (CST, #8242), 1:1000 Phospho-NF-κB p65 (CST, 4025), 1:1000 Phospho-JNK (Thr183/Tyr185, CST, #9255), 1:1000 JNK (Proteintech, 51151-1-AP), 1:1000 NLRP3 (Abcam, ab214185), 1:1000 ASC2 (Abcam, ab47092), 1:1000 IL-1 beta (Abcam, ab9722), 1:1000 IL-18 (Abcam, ab71495), 1:1000 pro-Caspase-1 + p10 + p12 (Abcam, ab179515), 1:1000 Cleaved Caspase-1 (CST, #89332), 1:1000 GSDMD (CST, 93709), 1:1000 Cleaved GSDMD (CST, 50928), 1:1000 AIM2 (Abcam, ab180665), 1:1000 p53 (Proteintech, 10442-1-AP), 1:1000 xCT (Proteintech, 26864-1-AP), 1:1000 FTH1 (Proteintech, 10727-1-AP), 1:1000 COX2 (Proteintech, 12375-1-AP), and 1:1000 GPX4 (Proteintech, 14432-1-AP). The membranes were washed with TBST for 10 min three times, and incubated with HRP-conjugated secondary antibody (CST, 7074 or 7076) at room temperature for 1 h. Membranes were then washed with TBST, and visualized using a chemiluminescence system (Bio-Rad, USA).

### Scanning electron microscopy (SEM)

LV tissues were isolated and rinsed with PBS. Then, ophthalmic scissors and a scalpel were used to cut into 1 mm × 1 mm × 1 mm pieces. Tissue was fixed in 2.5% glutaraldehyde over 2 h in room temperature and then was transferred to a 4 °C refrigerator. The fixed samples were then rinsed with PBS for 15 min three times and then transferred to 1% osmium PBS buffer for 2 h at room temperature. The ethanol gradient dewatering method was used for tissue dehydration. Samples were successively placed in 30–50–70–80–95–100–100% ethanol for 15 min. Finally, the samples were placed in isoamyl acetate for 15 min. Then, samples were placed into a critical point dryer for drying. Samples were pressed onto conductive carbon film with double-sided adhesive and placed on the sample table of the gold ion spraying instrument for about 30 s. Observation and image acquisition were performed under a scanning electron microscope.

### Transmission electron microscope (TEM)

LV tissue was isolated and rinsed with PBS. The method of fixation and dehydration was the same as described above for SEM. After dehydration, the mixture was permeated overnight with a 1:1 mixture of acetone and 812 embedding agents. Embedding was performed at 60 °C for 48 h, and slices were 60-nm-thick. Uranium-lead double staining (2% ur-acetate saturated water solution, lead citrate, 15 min each) was performed and sections were dried overnight at room temperature. Observation of morphological and structural changes in mitochondria was performed using a TEM for image collection and analysis.

### RNA extraction and RT-PCR

Total RNA was extracted from LV tissue of TAC mice and control mice or HL-1 mouse cardiac muscle cells (Procell CL-0605 were kindly provided by Procell Life Sciences & Technology Co., Ltd) using TRIzol™ reagent according to the manufacturer’s instructions (Sigma-Aldrich, Saint Louis, MO, USA). RNA was dissolved in sterile water and quantified by spectrophotometry at 260 nm, after which it was reverse-transcribed using an All-in-One cDNA Synthesis SuperMix (B24403, Bimake, Houston, TX, USA). RT-PCR was performed using ABI Prism v2.04 (Applied Biosystems, Foster City, CA, USA) using an ABI 7500 PCR instrument (Applied Biosystems) to determine the expression of specific genes using a SYBR Green qPCR Master Mix (B21202, Bimake, Houston, TX, USA). The PCR conditions were 1 min at 95 °C followed by 40 cycles of 95 °C for 15 s and 60 °C for 30 s, and then was 15 s for 95 °C, 60 °C for 1 min, and 15 s for 95 °C. The specific primers used to amplify genes are listed in Table 1. Relative amounts of mRNA for specific genes were calculated using 2^−ΔΔCt^ values. Each sample was run in duplicate, and the mean value of each set of duplicates normalized to that of mouse GAPDH was used to calculate relative gene expression. Experiments were carried out in triplicate.

**Table 1:** Primer sequence.

Gene	Forward sequence	Reverse sequence	Product length/bp
GAPDH	GGTTGTCTCCTGCGACTTCA	TGGTCCAGGGTTTCTTACTCC	183
IL-1β	TGCCACCTTTTGACAGTGATG	ATACTGCCTGCCTGAAGCTC	162
IL-18	GTTTACAAGCATCCAGGCACAG	GAAGGTTTGAGGCGGCTTTC	151
MCP-1	CAGGTCCCTGTCATGCTTCT	GTGGGGCGTTAACTGCATCT	91
MIP1α	CCATATGGAGCTGACACCCC	GAGCAAAGGCTGCTGGTTTC	101
ICAM1	TGTCAGCCACCATGCCTTAG	CAGCTTGCACGACCCTTCTA	132
CXCL1	ACTCAAGAATGGTCGCGAGG	GTGCCATCAGAGCAGTCTGT	123
CXCL2	TGCTGTCCCTCAACGGAAGA	CTCTCAGACAGCGAGGCAC	94
ANP	GCTTCGGGGGTAGGATTGAC	CACACCACAAGGGCTTAGGA	144
BNP	CGGATCCGTCAGTCGTTTGG	AAAGAGACCCAGGCAGAGTCA	100
MMP2	AACGGTCGGGAATACAGCAG	AAACAAGGCTTCATGGGGGC	123
MMP9	CCAGCCGACTTTTGTGGTCT	TGGCCTTTAGTGTCTGGCTG	212

In the miRNA experiment, the total RNA was reverse-transcribed using a miRNA cDNA Synthesis Kit (CoWin Biosciences, Jiangsu, China). RT-PCR also was performed using an ABI 7500 PCR instrument, and the expression of miR-351 was determined (sense primer: CTCCCTGAGGAGCCCTTTGAGC, antisense primer: provided by assay kit) using a miRNA PCR Assay Kit (CoWin Biosciences, Jiangsu, China). The PCR conditions were 10 min at 95 °C followed by 40 cycles of 95 °C for 15 s and 60 °C for 1 min. Data were normalized to levels of small nucleolar RNA (snRNA) U6 (sense primer: GGAACGATACAGAGAAGATTAGC, antisense primer: GGAACGCTTCACGAATTTGCG).

### Immunofluorescence analysis

Hearts tissues were isolated and rinsed with PBS and fixed in 4% PFA over 24 h. The hearts were then dehydrated and paraffin-embedded. Next, 5-µm-thick slices were cut for histological and immunochemical analyses. Paraffin sections were dewaxed with water, and antigens were retrieved by sodium citrate heating. Endogenous peroxidase was removed by adding 30% H_2_O_2_, and an immunohistochemical pen was used to draw a circle around the tissue. Subsequently, 5% goat serum was added to block the tissue. Sheep anti-mouse NLRP3 primary antibody (Abcam, ab179515), rabbit anti-mouse cTnt primary antibody (Abcam, ab179515), and wheat germ agglutinin (WGA) were diluted to a ratio of 1:100, dropped onto the sections, and incubated at room temperature for 60 min. Sections were then washed three times with PBS for 3 min. Anti-sheep and anti-rabbit secondary fluorescent antibodies were prepared at a ratio of 1:1000. Slices were incubated with secondary antibody at room temperature in the dark for 60 min, and then were washed with PBS three times for 3 min; 50 μL DAPI solution was added to the section for incubation for 5 min followed by three PBS washes. A fluorescence microscope was used to observe and collect images in a darkroom.

### TUNEL analysis

Heart tissues were isolated, rinsed with PBS, and fixed using the same method as described above. After the slices were shaken dry, the membrane-rupture working fluid was added to cover the tissue. Twenty minutes later, the membrane-rupture working fluid was discarded, and TUNEL dye was added onto the slices. Two hours later, DAPI was added to slices for 5 min, avoiding light. Slices were then washed with PBS three times for 3 min. The slices were shaken dry and sealed with antifluorescence quenching sealant. Fluorescence microscopy was performed in a darkroom for observation and image collection.

### ROS analysis

After anesthesia, heart tissue was separated, washed with PBS, and rapidly frozen in liquid nitrogen. The tissue was then embedded with an OCT embedding agent. After embedding, 6-µm-thick sections were generated with a constant temperature freezing microtome for ROS staining. After slightly drying the frozen sections, a tissue pen was used to draw a circle around the tissue. ROS dye was added to the circle and incubated at 37 °C for 30 min, avoiding light, followed by three 3 min PBS washes. After the sections were slightly dried, DAPI dye was added into the circle and incubated in the dark for 10 min at room temperature. Slices were then washed with PBS three times for 3 min/time. The slices were shaken dry and sealed with antifluorescence quenching sealant. Fluorescence microscopy was performed in a darkroom for observation and image collection.

### MDA, T-SOD, and GSH analysis

The contents of MDA (E-BC-K025-S, E-BC-K028-M, Elabscience Biotechnology Co., Ltd, Wuhan, China), T-SOD (E-BC-K028-M, Elabscience Biotechnology Co., Ltd, Wuhan, China), and GSH (E-BC-K030-M, Elabscience Biotechnology Co., Ltd, Wuhan, China) were determined by colorimetry. The assays were performed according to the manufacturer’s recommendations.

### Histological and immunochemical analysis

Heart tissue was isolated, rinsed with PBS, and fixed. The method of fixation and dehydration was the same as for immunofluorescence analysis. An UltraSensitive^TM^ SP (Mouse/Rabbit) IHC Kit (KIT-9710, MXB Biotechnologies, Fuzhou, China) was used to complete the experiment. After blocking with 5% goat serum and incubation with 1:100 Collagen I (ABclonal, A16699), Collagen III (Proteintech,22734-1-AP), fibronectin, and α-SMA (CST, 2118) at room temperature for 60 min, samples were washed with PBS three times for 3 min/time. Secondary antibody was added followed by incubation at room temperature for 10 min and three PBS washes for 3 min each. Streptomyces anti-biotin protein-peroxidase reagent was added and incubated at room temperature for 10 min, then washed with PBS three times for 3 min/time. Freshly prepared DAB reagent was added onto the section for color rendering. PBS rinsing stopped the color development, hematoxylin redyeing, 1% hydrochloric acid ethanol differentiation, and PBS rinsing cyanosis. Gradient dehydration and transparency with xylene. Seal with neutral gum. Optical microscopy was used to observe and capture images.

### Dual-luciferase reporter assay

Bioinformatics tools (http://www.targetscan.org/vert_72/) were used to predict microRNA binding sites in MLK3. A WT or mut-MLK3 fragment was constructed and inserted downstream of the luciferase reporter gene of the pMIR-REPORT plasmid (GenePharma, shanghai, China). 293T cells were transfected with empty pmirGLO-NC, pmirGLO-MLK3-WT, pmirGLO-MLK3-MUT, or positive control vectors. Then, 293T cells were cotransfected with the miR-351 mimic or miR-NC by using Lipofectamine 3000 (Invitrogen). The relative Firefly and Renilla luciferase activity were detected at 24 h after transfection.

### Statistics

Data are presented as mean ± standard deviation. The number of cells/experiments (*n*) or animal (*N*) studied per experiment is indicated. Statistical analyses were performed with Student’s *t* test, one-way ANOVA with the Tukey’s multiple comparison post-hoc test, or two-way ANOVA followed by post-hoc Fisher LSD test for multiple comparisons. Survival rate analysis was performed with Kaplan–Meier curve method. Analyses were carried out with Prism 7 (GraphPad, San Diego, CA, USA) and SPSS v19.0 (IBM, Armonk, NY, USA). *P* < 0.05 was considered significant.

## Results

### MLK3 inhibitor URMC-099 attenuates cardiac dysfunction after TAC

**Fig. 1:**
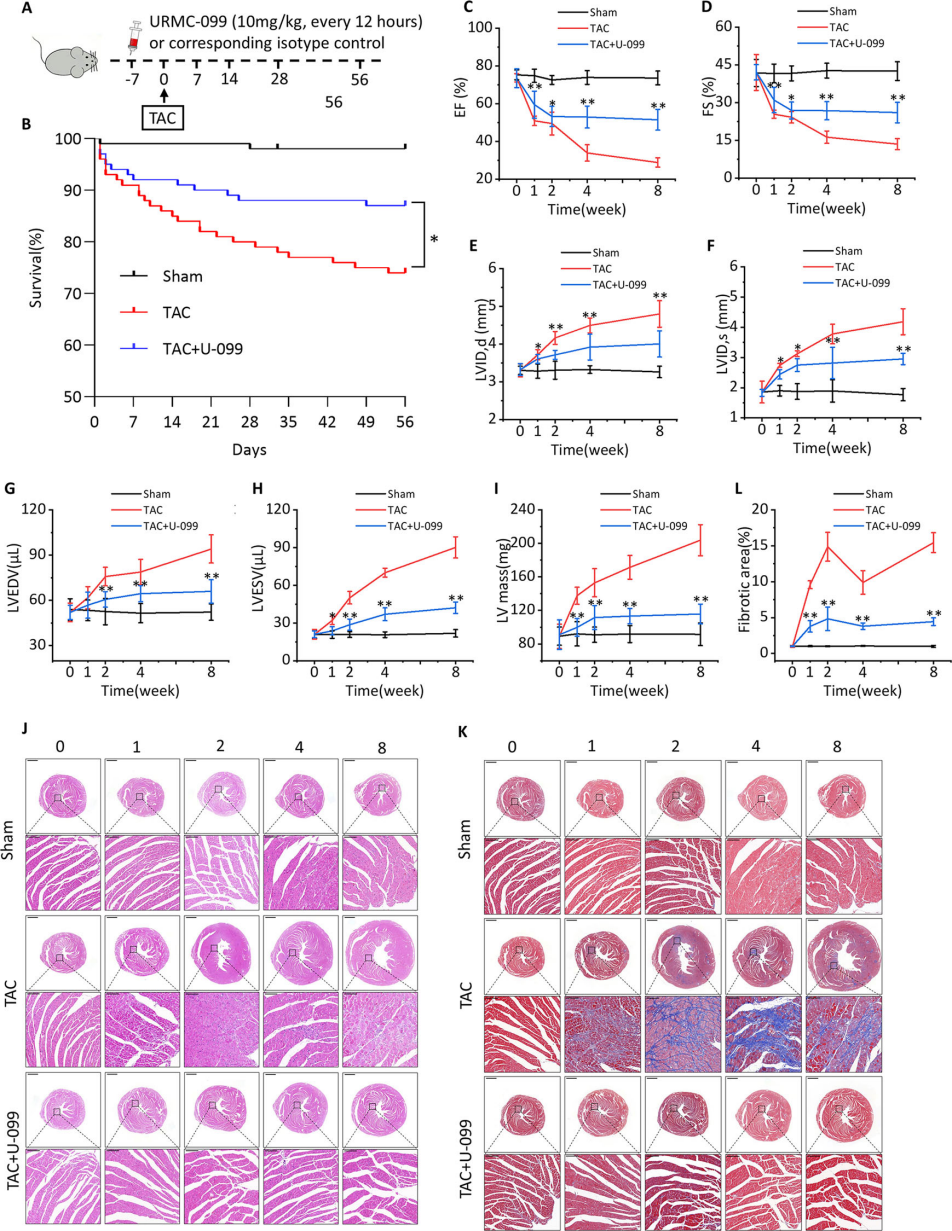
Inhibition of MLK3 improves cardiac function induced by pressure overload. **a** Schematic outline of experiments performed in panels. Mice were in administration of URMC-099 (10 mg/kg, i.p, every 12 h, 7 days before TAC surgery until 56 days after TAC) or isotype. Sham group was injected with isotype control. **b** Kaplan–Meier survival plots of different groups sham, TAC and TAC + U-099 subjected to TAC at different time points. EF% (**c**), FS% (**d**), LVID; d (**e**), LVID; s (**f**), LVEDV (**g**), LVESV (**h**), and LV mass (**i**) of TAC, TAC + U-099, or sham mice after 1, 2, 4, and 8 weeks, respectively. Mean ± SEM, *n* = 5 biologically independent samples, *P < 0.05, **P < 0.01 vs TAC by one-way ANOVA followed by Tukey’s multiple comparisons test. **j**–**l** H&E staining (left panel) and Masson’s trichrome staining (right panel) of hearts from TAC, TAC + U-099, or sham mice after 1, 2, 4, and 8 weeks, respectively. Mean ± SEM, *n* = 3 biologically independent samples, the lower scale bar indicates 1 mm, and the higher scale bar indicates 100 μm, **P < 0.01 vs TAC by one-way ANOVA followed by Tukey’s multiple comparisons test.

To determine whether inhibition of MLK3 expression improved cardiac function in TAC mice, we examined the time course of cardiac function in response to URMC-099. Mice were given i.p. injections of URMC-099 7 days before the modeling to ensure low MLK3 expression levels (Fig. 1a). Results showed that URMC-099 treatment led to reduction of protein relative expression level of MLK3, p-MLK3, JNK, and p-JNK, but no changes in p-MLK3/MLK3 and p-JNK/JNK (Fig. S1). There were no baseline differences in ventricular structure or function between mice treated with URMC-099 and without it. Eight weeks after TAC, about only 10% of URMC-099 treated mice died, while about 25% of control mice died (Fig. 1b). We also observed changes in cardiac function in TAC mice at week 1, 2, 4, and 8, but not in sham mice (Fig. 1c–i). Compared with sham mice, echocardiography showed significant worsening cardiac function at week 1 and 2 in TAC and URMC-099 mice, but URMC-099 mice performed better. However, the cardiac function of TAC mice remained at a significantly reduced level of cardiac function, while URMC-099 mice maintained a stable level, showing a significant increase in LVEF and LVFS, and a significant decrease in LVID; d, LVID; s, LVEDV, LVESV, and LV mass compared with TAC mice at week 4 and 8 (Fig. 1c–i).

### The MLK3 inhibitor URMC-099 inhibits cardiac hypertrophy and collagen deposition in TAC mice

To determine whether URMC-099 could ameliorate cardiac hypertrophy and collagen deposition after TAC, we analyzed cardiomyocyte size and collagen deposition of cardiac sections by HE & Masson staining. Results showed that cardiomyocyte size and collagen deposition in sham mice did not change over time, while in TAC mice they were significantly increased. Compared with TAC mice, the changes in cardiomyocyte size and collagen deposition were significantly reduced in URMC-099 mice (Fig. 1j–l).

### TAC activates MLK3 signaling to induce pyroptosis- and ferroptosis-related protein expression

**Fig. 2:**
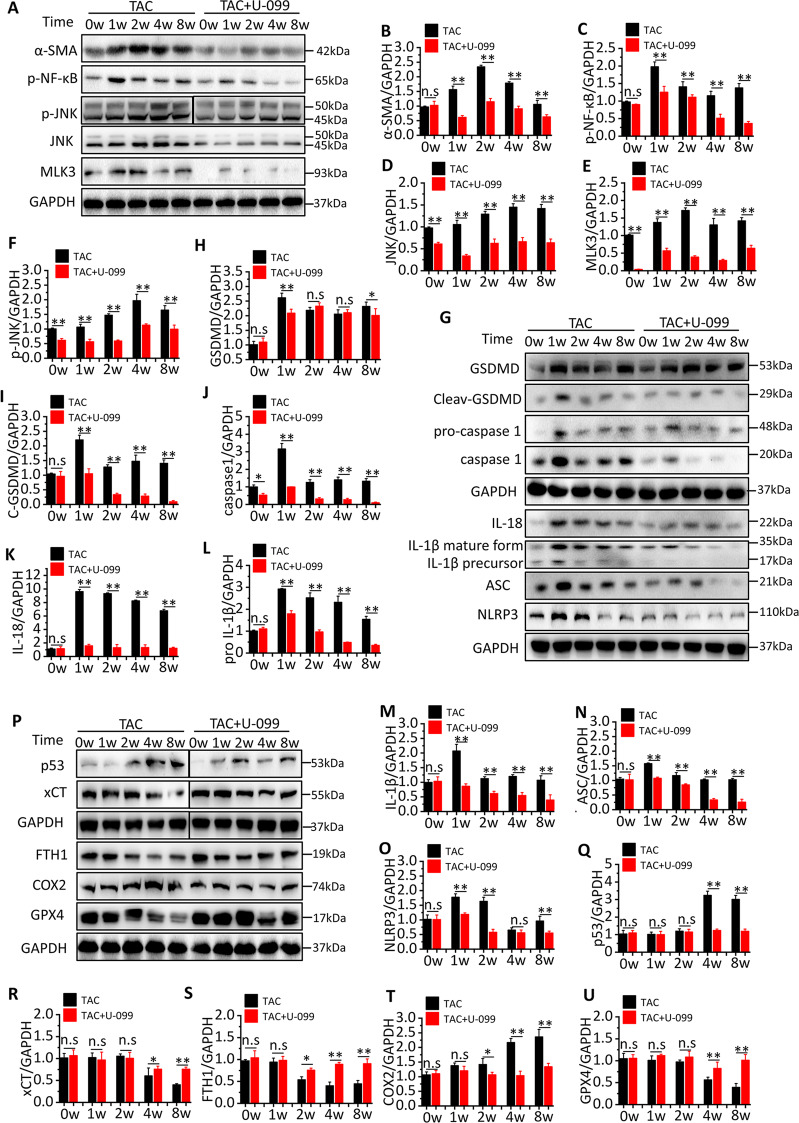
Inhibition of MLK3 alters NF-κB and JNK signaling pathway. **a**–**f** Representative western blotting and quantification of MLK3, JNK, NF-κB, α-SMA, and GAPDH served as a loading control. *n* = 3 for each group. **g**–**o** Representative western blotting and quantification of inflammation and pyroptosis-related proteins, including GSDMD, cleaved GSDMD, pro-caspase-1, caspase-1, IL-18, pro-IL1β, IL-1β, ASC, NLRP3, and GAPDH served as a loading control. *n* = 3 for each group. **p**–**u** Representative western blotting and quantification of JNK downstream protein, including p53, xCT, GPX4, and ferroptosis-related proteins, including COX2 and FTH1. GAPDH served as a loading control. *n* = 3 for each group. Mean ± SEM, control values were set to 1. *P < 0.05, **P < 0.01 vs TAC + U-099 by Student’s *t* test.

To investigate whether TAC had an effect on MLK3 expression, we detected the expression of major proteins downstream of MLK3, including NF-κB and JNK. The results showed that the expression of MLK3 was significantly increased over time in TAC mice, but it almost completely disappeared in URMC-099 mice. The expression of phosphorylation NF-κB was significantly increased in week 1, and gradually decreased after the week 2, reaching its lowest level in the week 8. The expression of JNK and phosphorylation JNK increased gradually and reached its highest level in the week 8. Compared with TAC mice, the expression of phosphorylation NF-κB, JNK and phosphorylation JNK were significantly decreased in URMC-099 mice. α-SMA expression was also significantly decreased in them (Fig. 2a–f).

In order to further clarify the specific mechanism by which MLK3 and its downstream proteins promote myocardial fibrosis, we detected the expression of inflammatory response-related proteins induced by NF-κB as well as oxidative damage related proteins induced by JNK at different time points. The results showed that the expression of downstream proteins of NF-κB, including NLRP3, ASC, IL-18, IL1β, caspase-1, and GSDMD, was significantly elevated after TAC and peaked in week 1. The expression of NLRP3 and ASC gradually declined after the week 2, and reached normal levels in the week 4. However, the expression of GSDMD hit another peak in week 8. In contrast, compared with TAC mice, URMC-099 effectively reduced the expression of NLRP3, ASC, IL-18, IL1β, caspase-1, and GSDMD, especially in the week 1 (Fig. 2g–o). Detection of proteins downstream of JNK in TAC mice revealed that the expression of p53 and COX2 was significantly increased and peaked in week 8, whereas the expression of xCT, GPX4, and FTH1 was significantly decreased and reached their lowest levels in week 8. However, compared with TAC mice, URMC-099 successfully inhibited changes in p53, COX2, xCT, GPX4, and FTH1 (Fig. 2p–u).

### MLK3 depletion reverses cardiac dysfunction and pyroptosis levels after 1 week of TAC

**Fig. 3:**
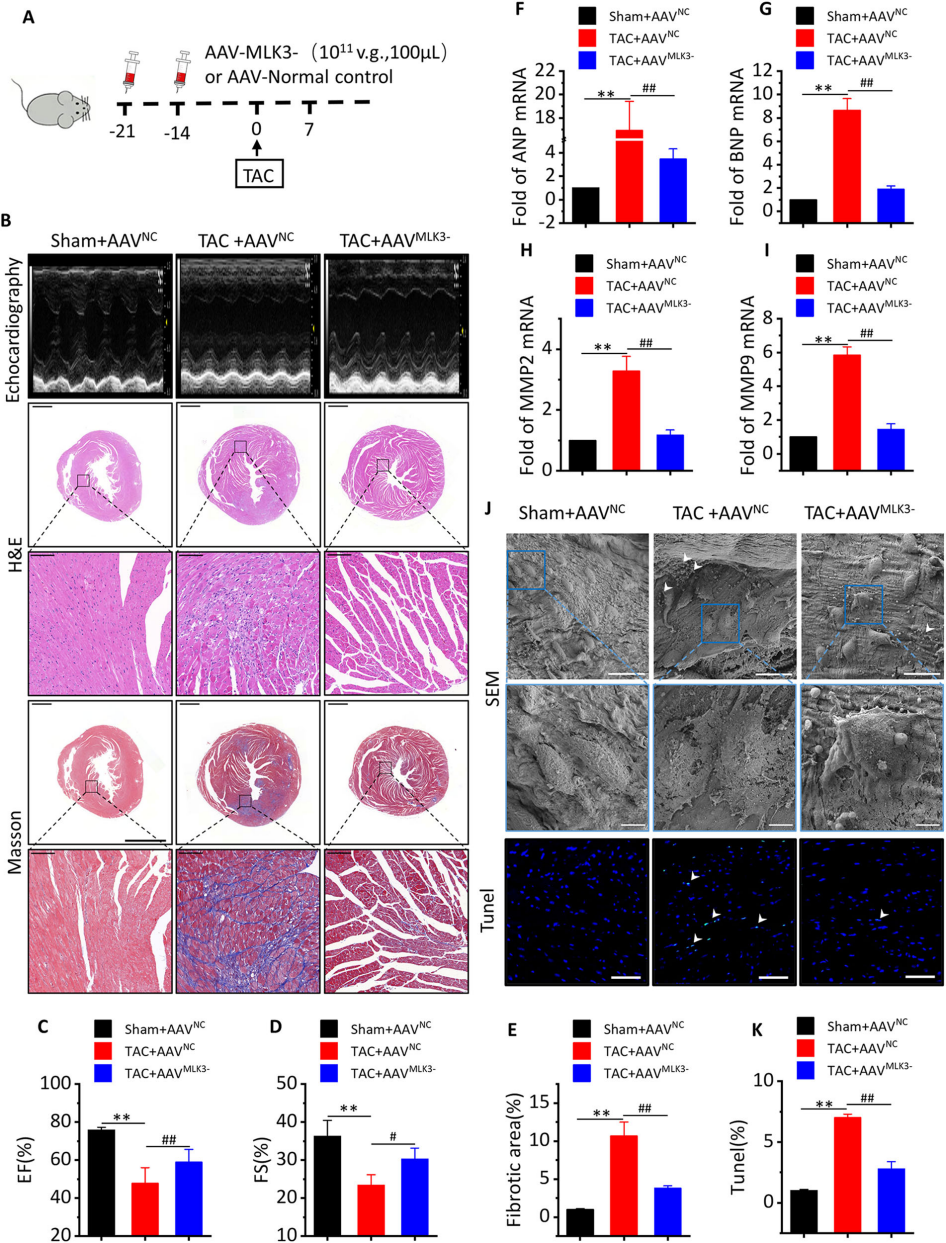
Inhibition of MLK3 improves cardiac function and inhibits pyroptosis in day 7 of TAC. **a** Schematic outline of experiments performed in panels. Mice were in administration of AAV-MLK3 (10^11^ v.g.,100 μL, 14 and 21 days before TAC surgery, until 7 days after TAC) or AAV-NC. Sham group was injected AAV-NC. **b** Representative M-mode echocardiography recordings (upper row), H&E stained sections of heart (middle row) and sections of Masson’s trichrome-stained heart tissue (middle row)), quantitative analysis of the collagen area/left ventricular (below Masson’s trichrome staining) in Sham + AVV^NC^, or TAC + AVV^NC^ or TAC + AVV^MLK3-^ mice after 1 week. the lower scale bar indicates 1 mm, and the higher scale bar indicates 100μm. **c–e** Echocardiographic parameters: EF% (**c**), FS% (**d**) and fibrotic area (**e**) of Sham + AVV^NC^, TAC + AVV^NC^ or TAC + AVV^MLK3−^ mice after 1 week. *n* = 5 for each group. Mean ± SEM, fibrotic area control values were set to 1. **P < 0.01 vs sham-AVV^NC^, ^#^*P* < 0.05, ^##^*P* < 0.01 vs TAC + AVV^NC^ by one-way ANOVA followed by Tukey’s multiple comparisons test. Transcript level of ANP (**f**) and BNP (**g**), as determined by qRT-PCR. Transcript level of MMP2 (**h**) and MMP9 (**i**), as determined by qRT-PCR. *n* = 5. Mean ± SEM, **P < 0.01 vs Sham + AVV^NC^, ^##^*P* < 0.01 vs TAC + AAV^NC^ by one-way ANOVA followed by Tukey’s multiple comparisons test. **j** Representative images of SEM (upper row, the lower SEM scale bar indicates 15 µm, and the higher SEM scale bar indicates 5 µm) and dead cardiomyocytes in a section of mice heart measured by TUNEL staining (bottom row) from Sham + AVV^NC^, TAC + AVV^NC^, or TAC + AVV^MLK3^ mice after 1 week. Green staining (see white arrows) indicates dead cells (blue, nucleus. Scale bar, 100 μm). **k** Quantification of TUNEL assay, *n* = 5. Mean ± SEM, **P < 0.01 vs Sham + AVV^NC^, ^##^*P* < 0.01 vs TAC + AAV^NC^ by one-way ANOVA followed by Tukey’s multiple comparisons test.

To demonstrate the effect of MLK3 on the regulation of NF-κB and pyroptosis, mice were subjected to an i.v. injection of AAV^MLK3−^ (Fig. 3a). Western blot analysis showed the mice had a nearly complete loss of MLK3 after 21 days i.v. injection of AAV^MLK3−^ (Fig. S1). Compared with Sham + AAV^NC^ mice, LVEF, and LVFS were significantly decreased, while LVID; d and LVID; s, LVEDV, LVESV, and LV mass were significantly increased in TAC + AAV^NC^ mice. In contrast, compared with TAC + AAV^NC^ mice, the cardiac function was significantly improved in TAC + AAV^MLK3−^ mice (Figs. 3b–d and S2). Accordingly, TAC-induced increases of Nppa and Nppb (Fig. 3f, g) were partly alleviated in TAC + AAV^MLK3−^ mice compared with TAC + AAV^NC^ mice, as well as TAC-induced increases in MMP2 and MMP9 (Fig. 3h, i). The HE results showed that the hearts were significantly enlarged in TAC + AAV^NC^ mice, whereas hearts from AAV^MLK3−^ mice were not significantly enlarged after TAC (Fig. 3b). The Masson results showed that TAC + AAV^NC^ mice had more obvious blue collagen deposition in the heart compared with that in TAC + AAV^MLK3−^ mice (Fig. 3b, e). To determine whether pyroptosis occurred in TAC hearts, and whether it was closely related to MLK3, the SEM results showed more inflammasomes and membrane rupture in TAC + AAV^NC^ mice, membrane were completely ruptured in the stage of late pyroptosis in the improved pictures, while more apoptotic cells were found in TUNEL staining in TAC + AAV^NC^ mice compared with those in AAV^MLK3−^ mice (Fig. 3j, k).

### MLK3 depletion inhibits pyroptosis-related protein expression and the levels of inflammation cytokines after 1 week of TAC

**Fig. 4:**
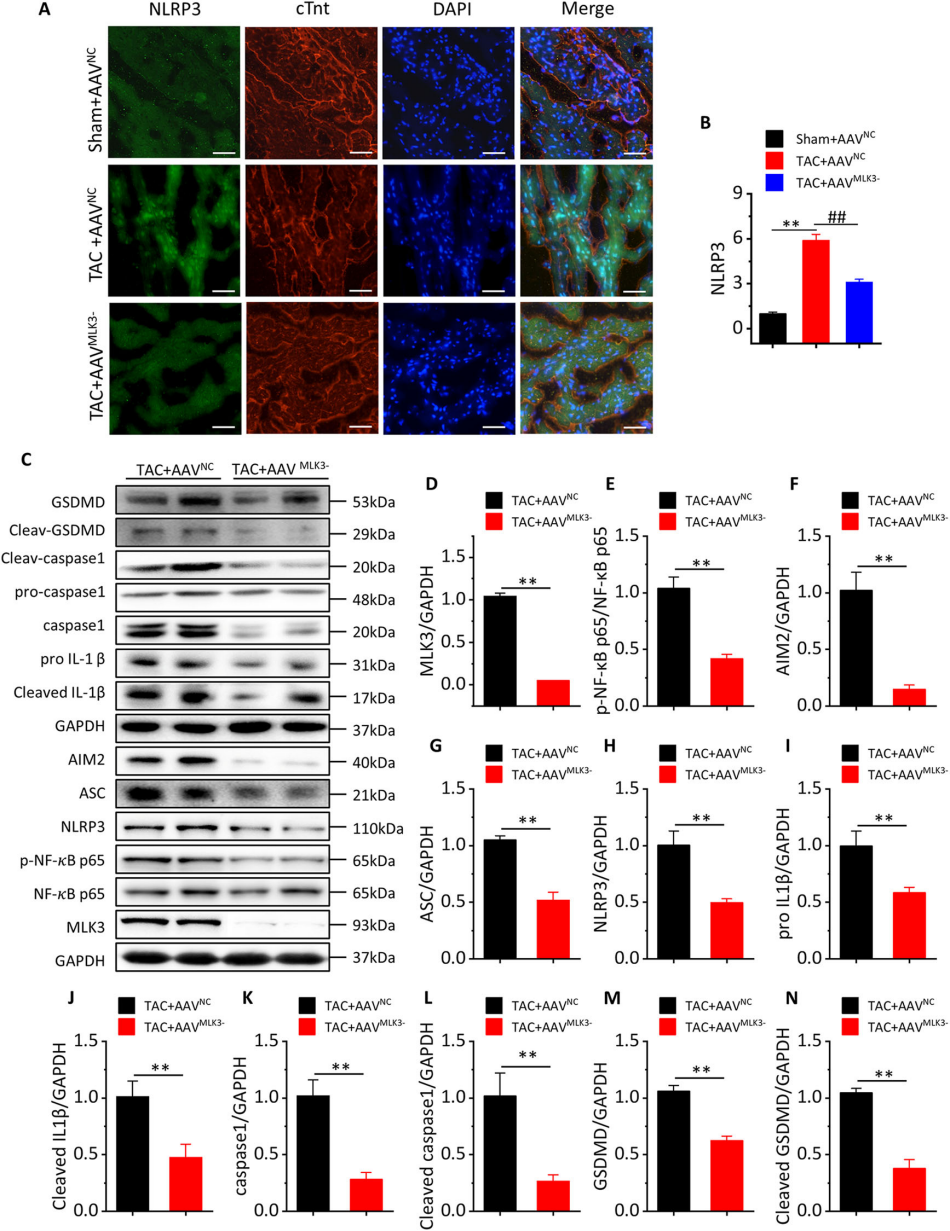
Pyroptosis and inflammation-related proteins and genes are significantly downregulated after inhibition of MLK3 in day 7 of TAC. **a** Representative image of cardiac section stained with NLRP3 antibody (in green) for inflammasome, cTnt antibody (in red) for cardiomyocyte, and DAPI (in blue) for nucleus, obtained at day 7 of TAC, scale bar 100 µm. **b** Quantification of immunofluorescence analysis, *n* = 3. Mean ± SEM, **P < 0.01 vs Sham + AVV^NC^, ^##^*P* < 0.01 vs TAC + AAV^NC^ by one-way ANOVA followed by Tukey’s multiple comparisons test. **c**–**n** Representative western blotting and quantification of inflammation and pyroptosis-related proteins, including GSDMD, cleaved GSDMD, pro-caspase-1, caspase-1, IL-18, pro-IL1β, IL-1β, AIM2, ASC, NLRP3, NF-κB p65, p-NF-κB p65, MLK3, and GAPDH served as a loading control. *n* = 4 for each group. Mean ± SEM, control values were set to 1. *P < 0.05, **P < 0.01 vs TAC + AAV^NC^ by Student’s *t* test.

In order to clarify the regulatory effect of MLK3 on pyroptosis, we measured the expression of pyroptosis-related proteins and the levels of inflammation cytokines. We found that NLRP3 was significantly increased in cardiomyocytes, especially in the nucleus in TAC + AAV^NC^ mice. However, NLRP3 was significantly inhibited in TAC + AAV^MLK3−^ mice (Fig. 4a, b). Compared with TAC + AAV^NC^ mice, the levels of MLK3, p-NF-κB p65, NLRP3, ASC, AIM2, pro-IL1β, cleaved IL1β, caspase-1, cleaved caspase-1, GSDMD, and cleaved GSDMD were significantly decreased in TAC + AAV^MLK3−^ mice (Fig. 4c–n). Inflammation-related mRNA expression was detected by RT-PCR, showing that expression of IL1β and IL-18 was significantly increased in TAC + AAV^NC^ mice and inhibited by AAV^MLK3−^ (Fig. S3a, b). We also found that monocyte chemoattractant protein 1 (MCP-1), ICAM1, macrophage inflammatory protein 1 alpha (MIP1α), CX-C motif ligand 1 (CXCL1), and CX-C motif ligand 2 (CXCL2) mRNA were activated by TAC and significantly decreased in TAC + AAV^MLK3−^ mice (Fig. S3).

Meanwhile, we cultured HL-1 cells by means of inhibiting MLK3. Our data showed that MLK3 was upregulated when HL-1 cells were treated with lipopolysaccharide (LPS) (Fig. S4a, b). Similarly, we found that pyroptosis-related protein expression, like NLRP3 and GSDMD (Fig. S4a, c, d), the levels of inflammation cytokines, such IL-18, IL-1β, the mRNA level of Nppa and Nppb, and the activity of caspase-1 were significantly increased. However, these levels were partly diminished when we suppressed MLK3 and inhibited of NLRP3 by MCC950 (Fig. S4e–i).

### MLK3 depletion reverses cardiac dysfunction and ferroptosis levels after 8 weeks of TAC

**Fig. 5:**
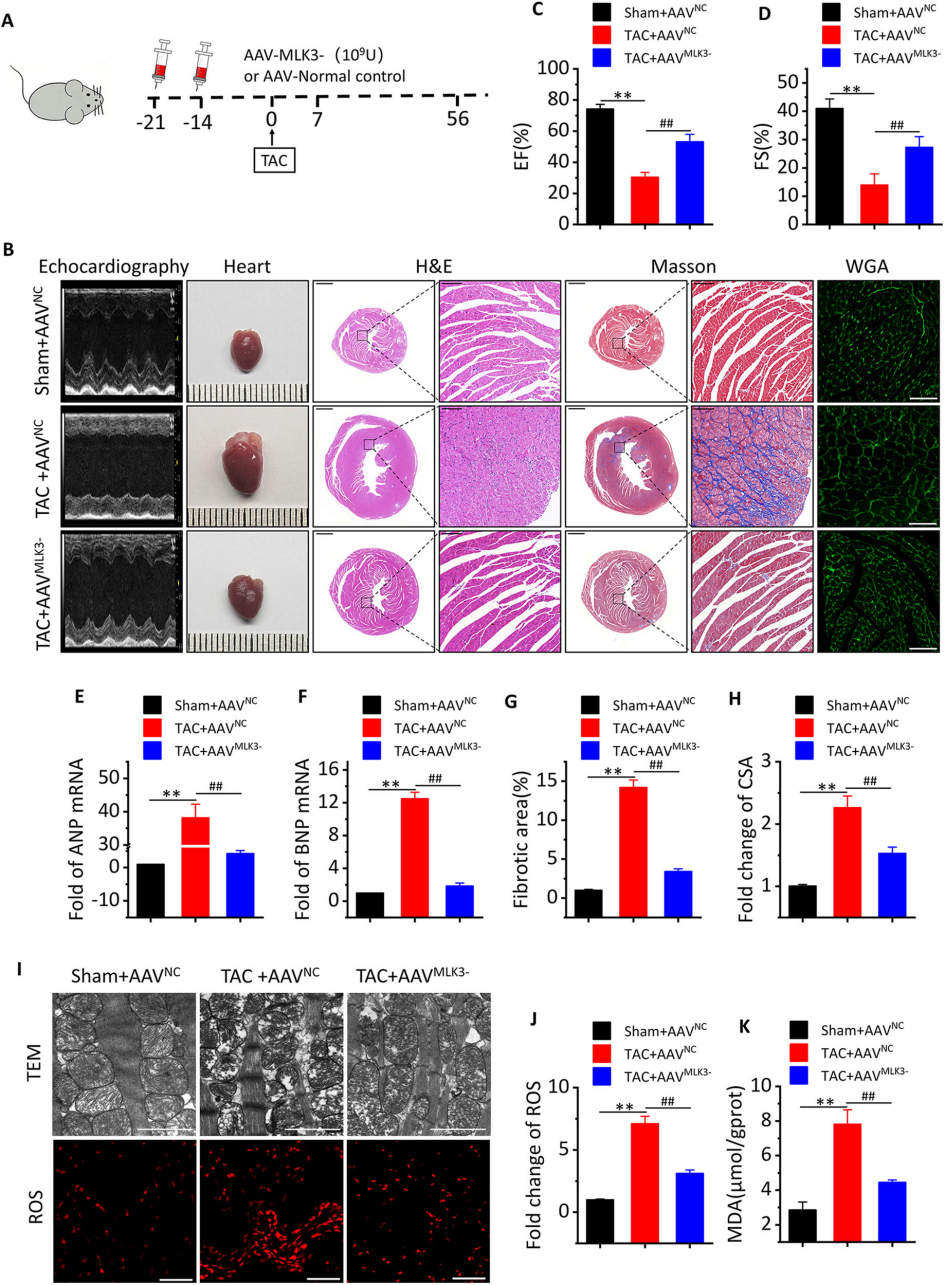
Inhibition of MLK3 improves cardiac function and inhibits ferroptosis at week 8 of TAC. **a** Schematic outline of experiments performed in panels. Mice were in administration of AAV-MLK3 (10^11^ v.g.,100 μL, 14 and 21 days before TAC surgery, until 8 weeks after TAC) or AAV-NC. Sham group was injected with AAV-NC. **b** Representative M-mode echocardiography recordings (upper row), heart sizes, H&E stained sections of heart (middle row), sections of Masson’s trichrome-stained heart tissue (middle row), and representative images and analysis of wheat germ agglutinin staining of the heart sections. Quantitative analysis of the collagen area/left ventricular (below Masson’s trichrome staining) and cross-sectional area (CSA) of myocyte in Sham + AVV^NC^, TAC + AVV^NC^, or TAC + AVV^MLK3−^ mice after 1 week. The lower scale of H&E and Masson bar indicates 1 mm, and the higher scale bar of H&E and Masson indicates 100 μm, WGA scale bar indicated 50 µm. **c**, **d** Echocardiographic parameters: EF% (**c**), FS% (**d**), fibrotic area (**g**), and CSA (**h**) of Sham + AVV^NC^, TAC + AVV^NC^, or TAC + AVV^MLK3−^ mice after 8 weeks. *n* = 5 for each group. Mean ± SEM, fibrotic area, and CSA control values were set to 1. **P < 0.01 vs Sham + AVV^NC^, ^#^*P* < 0.05, ^##^*P* < 0.01 vs TAC + AVV^NC^ by one-way ANOVA followed by Tukey’s multiple comparisons test. **e**, **f** Transcript level of ANP and BNP, as determined by qRT-PCR. **i** Representative images of TEM (upper row, the scale bar indicates 2 µm) and ROS in a section of mice heart measured by immunofluorescence staining (bottom row, red, ROS. Scale bar, 100 μm.) from Sham + AVV^NC^, TAC + AVV^NC^, or TAC + AVV^MLK3^ mice after 8 weeks. **j** Quantification of ROS assay, *n* = 5. Mean ± SEM, **P < 0.01 vs Sham + AVV^NC^, ^##^*P* < 0.01 vs TAC + AAV^NC^ by one-way ANOVA followed by Tukey’s multiple comparisons test. **k** MDA in whole ventricular lysates as measured by colorimetric method, *n* = 5. Mean ± SEM, **P < 0.01 vs Sham + AVV^NC^, ^##^*P* < 0.01 vs TAC + AAV^NC^ by one-way ANOVA followed by Tukey’s multiple comparisons test.

To demonstrate the effect of MLK3 on the regulation of JNK and ferroptosis, mice were subjected to an i.v. injection of AAV^MLK3−^ (Fig. 5a). Compared with Sham + AAV^NC^ mice, LVEF and LVFS were significantly decreased, while LVID; d and LVID; s, LVEDV, LVESV, and LV mass were significantly increased in TAC + AAV^NC^ mice. In contrast, compared with TAC + AAV^NC^ mice, the cardiac function was significantly improved in TAC + AAV^MLK3−^ mice. (Figs. 5b–d and S5a–e). Furthermore, TAC-induced increases of Nppa and Nppb (Fig. 5e, f) were partly alleviated in TAC + AAV^MLK3−^ mice compared with TAC + AAV^NC^ mice. In addition, HE staining showed that the heart was significantly enlarged, and that cardiac enlargement in AAV^MLK3−^ mice was not significant after TAC (Fig. 5b). Masson staining results showed that TAC + AAV^NC^ mice had obvious collagen deposition in the heart, which was significantly improved in TAC + AAV^MLK3−^ mice (Fig. 5b, g). WGA staining found that compared with Sham + AAV^NC^ mice, myocyte cross-sectional area in TAC + AAV^NC^ mice was significantly increased, and compared with TAC + AAV^NC^ mice, cell hypertrophy in TAC + AAV^MLK3−^ mice was significantly reduced (Fig. 5b, h). The TEM data showed that compared with Sham + AAV^NC^ mice, the mitochondria were irregularly arranged, smaller, hand ad thicker membranes and a darker color in TAC + AAV^NC^ mice. In addition, higher level of ROS was observed in TAC + AAV^NC^ mice. However, AAV^MLK3−^ reversed the changes in mitochondria and ROS caused by TAC (Fig. 5i, j). Meanwhile, we assessed levels of MDA, SOD, and GSH. These proteins are closely related to oxidative stress, and the results showed that compared with Sham + AAV^NC^ mice, the level of MDA (Fig. 5k) was significantly increased while the levels of SOD and GSH were significantly decreased in TAC + AAV^NC^ mice. Compared with TAC + AAV^NC^ mice, the level of MDA was significantly decreased and the levels of SOD and GSH were significantly increased in TAC + AAV^MLK3−^ mice (Figs. 5k and S5f, g).

### MLK3 depletion inhibits ferroptosis- and oxidative stress-related protein expression and myocardial fibrosis after 8 weeks of TAC

**Fig. 6:**
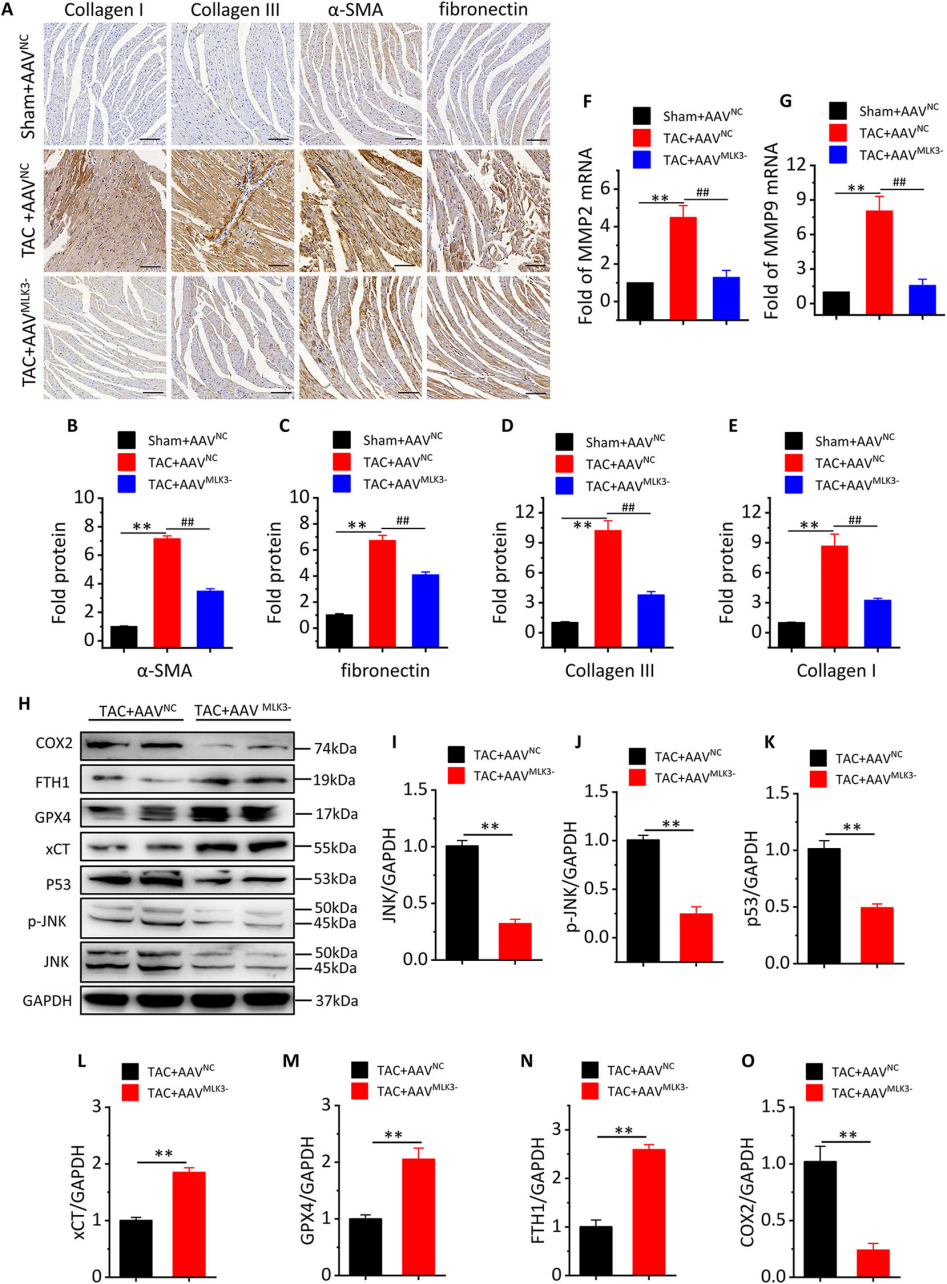
Fibrosis, oxidative stress and ferroptosis-related proteins are significantly downregulated after MLK3 was inhibited at day 7 of TAC. **a** Representative image of cardiac section stained with Collagen I, Collagen III, α-SMA, and fibronectin by IHC, obtained at week 8 of TAC, scale bar 100 µm. **b**–**e** Quantification results of Collagen I, Collagen III, α-SMA, and fibronectin, *n* = 3. Mean ± SEM, control values were set to 1, **P < 0.01 vs Sham + AVV^NC^, ^##^*P* < 0.01 vs TAC + AAV^NC^ by one-way ANOVA followed by Tukey’s multiple comparisons test. Transcript level of ANP (**f**) and BNP (**g**), as determined by qRT-PCR. **h**–**o** Representative western blotting and quantification of JNK, p53, xCT, GPX4, FTH1, and COX2 proteins and GAPDH served as a loading control. *n* = 4 for each group. Mean ± SEM, control values were set to 1. *P < 0.05, **P < 0.01 vs TAC + AAV^NC^ by Student’s *t* test.

To clarify the effect of MLK3 on ventricular remodeling, we found that Collagen I, Collagen III, fibronectin, and α-SMA expressions were significantly increased in TAC + AAV^NC^ mice after week 8, and that AAV^MLK3−^ reversed the changes (Fig. 6a–e). Meanwhile, we also found expressions of MMP2 and MMP9 (Fig. 6f, g) were significantly increased in TAC + AAV^NC^ mice, and significantly decreased in TAC + AAV^MLK3−^ mice compared with TAC + AAV^NC^ mice. To clarify the regulatory effect of MLK3 on ferroptosis, we detected ferroptosis- and oxidative stress-related protein expression. Compared with TAC + AAV^NC^ mice, the expressions of COX2, JNK, and p53 were significantly decreased, and expressions of xCT, GPX4, and FTH1 were significantly increased in TAC + AAV^MLK3−^ mice (Fig. 6h–o). We also observed the change of protein or mRNA with the intervention of FIN56 (HY-103087, MedChemExpress, Shanghai, China), a specific ferroptosis inducer, in HL-1 cells by means of inhibiting MLK3. Our data showed that the expression of MLK3, p-JNK, p53, and COX2 protein levels, the mRNA level of Nppa and Nppb, and MDA level were significantly increased, and FTH1 protein level and T-SOD and SGH levels were significantly decreased when HL-1 cells were treated with FIN56. However, these related biomarkers were partly reversed when we suppressed MLK3 (Fig. S6).

### miR-351 inhibits MLK3 expression to improve cardiac function

**Fig. 7:**
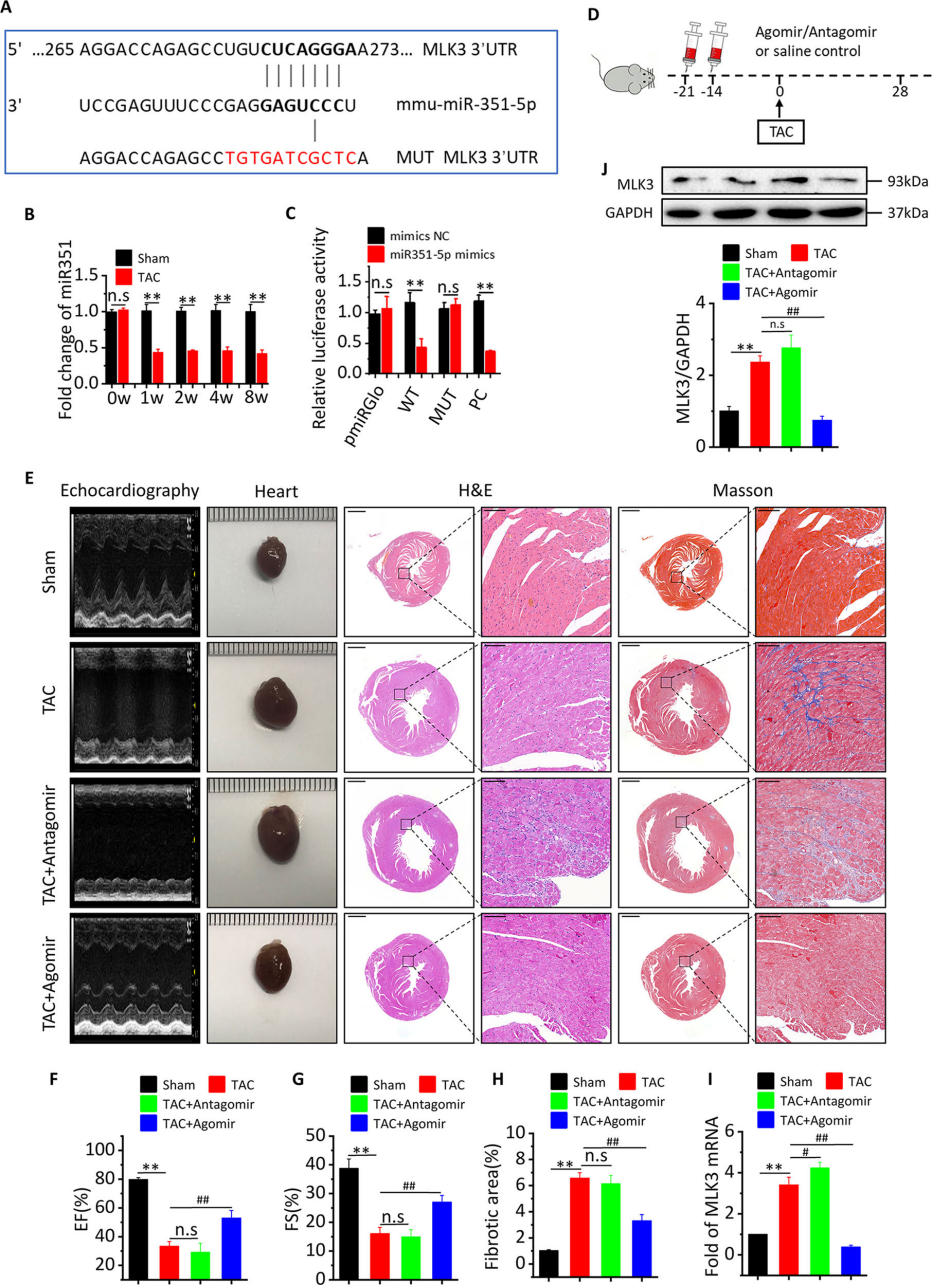
miR-351 prevents pressure overload-induced heart failure by MLK3 signaling. **a** Schematic illustration of the hypothetical duplexes formed by miR-351 with the 3′ 31 UTR of MLK3. **b** Expression of miR-351 in whole ventricular lysates as measured by qPCR after 1, 2, 4, and 8 weeks, respectively. normalized for the internal control U6 and expressed as fold increase over sham. *n* = 3. Mean ± SEM, **P < 0.01 vs Sham by Student’s *t* test. **c** Luciferase activities as quantified from the 293T cotransfected with the WT or mutant 3′ UTR of MLK3 luciferase reporter plasmids together with miR-351 inhibitor or mimic and or the corresponding control. Mean ± SD. **P < 0.01 by two-way ANOVA. **d** Schematic outline of experiments performed in panels. Mice were in administration of antagomir or agomir (14 and 21 days before TAC surgery, until 8 weeks after TAC). Sham and TAC group were injected with saline. **e** Representative M-mode echocardiography recordings (upper row), heart sizes, H&E sections (middle row), sections of Masson’s trichrome-stained heart tissue (middle row), quantitative analysis of the collagen area/left ventricular (below Masson’s trichrome staining) of myocyte in Sham, TAC, TAC + antagomir, or TAC + agomir mice after 4 weeks. The lower scale bar indicates 1 mm, and the higher scale bar indicates 100 μm. Echocardiographic parameters: EF% (**f**), FS% (**g**), and fibrotic area (**h**) of Sham, TAC, TAC + antagomir, or TAC + agomir mice after 4 weeks. *n* = 5 for each group. Mean ± SEM, fibrotic area control values were set to 1. **P < 0.01 vs Sham, ^##^*P* < 0.01 vs TAC by one-way ANOVA followed by Tukey’s multiple comparisons test. MLK3 mRNA (**i**) and protein (**j**) in whole ventricular lysates as measured by qPCR and western blot, respectively, normalized for the internal control GAPDH and expressed as fold increase over sham. *n* = 5. Mean ± SEM, **P < 0.01 vs Sham, ^##^*P* < 0.01 vs TAC by one-way ANOVA followed by Tukey’s multiple comparisons test.

To identify the potential mechanisms by which MLK3 functions in the context of CHF, we used the Targetscan.org platform to predict potential microRNAs targeting MLK3. The results showed that miR-351 may be a potential microRNA that could inhibit MLK3 expression (Fig. 7a). To determine whether the expression of miR-351 was changed in CHF mice, we detected the expression of miR-351 by RT-PCR. Compared with sham mice, the expression of miR-351 was significantly decreased in TAC mice at week 1, 2, 4, and 8 (Fig. 7b). In order to verify whether that miR-351 could interact with MLK3 and inhibit its expression, we constructed plasmids expressing WT and mutant MLK3, and used the dual-luciferase report gene system to determine MLK3 expression. The results showed that miR-351 could significantly inhibit the expression of MLK3 (Fig. 7a, c).

To clarify whether miR-351 plays an important role in the pathogenesis of CHF, we inhibited or enhanced the expression of miR-351 by treating mice with the antagomir or agomir of miR-351 before TAC induction (Fig. 7d). We found that LVID; d, LVID; s, LVEDV, LVESV, LV mass, heart size, and collagen deposition were significantly increased, whereas LVEF and LVFS were significantly decreased in TAC and antagomir mice compared with sham mice. Compared with TAC mice, LVID; d, LVID; s, LVEDV, LVESV, LV mass, heart size, and collagen deposition were significantly decreased, while LVEF and LVFS were significantly increased in agomir mice (Figs. 7e-h and S7a–e). We also detected the expressions of MLK3 protein and mRNA, and the results showed that miR-351 agomir effectively inhibited the expression of MLK3 protein and mRNA (Fig. 7i–j). Accordingly, TAC-induced increases of Nppa and Nppb (Fig. S7f, g) were partly alleviated in agomir mice compared with antagomir mice, as well as TAC-induced increases of MMP2 and MMP9 (Fig. S7h, i).

## Discussion

In our present study, we investigated the role of MLK3 in the development of CHF. We found that inhibition of MLK3 can effectively improve cardiac function, prevent myocardial fibrosis, and prevent hypertrophy in TAC mice. In addition, our data showed that the regulatory mechanism can be attributed to inhibiting NF-κB/NLRP3-mediated inflammation and pyroptosis in the early stage of pressure overload, while the effect of MLK3 inhibition at the end stage of pressure overload is mainly mediated by limiting oxidative damage and ferroptosis mediated by the JNK/p53 signaling pathway. Meanwhile, we noticed that miR-351 negatively regulated the expression of MLK3, a direct target of miR-351b. Overexpression of miR-351 can partly alleviate heart failure in response to TAC in vivo. Our data demonstrated that miR-351 may be a crucial regulator of murine CHF for first time, thus providing useful insights into the utility of the mir-351/MLK3 pathway as a diagnostic and therapeutic target for CHF.

It has recently been reported that MLK3 is involved in many disease processes, including tumor formation and metastasis, apoptosis, and cerebral ischemic injury. Further, it has been shown that MLK3 mediates neuronal damage due to activation of microglia^38^. CHF has a similar characteristic that the activation of fibroblasts will produce harmful effects on cardiac myocytes. Calamaras et al.^42^ reported increased MLK3 expression in human patients with cardiomyopathy. Recently, a report suggested that MLK3-deficient mice are protected against diet-induced NASH and liver fibrosis^43^. Lin et al. also reported that MLK3 mediates fibroblast activation to cause pulmonary fibrosis^44^. Another study revealed its potential role for treating invasive breast cancer^45^. Therefore, we hypothesized that MLK3 mediates myocardial fibrosis. In our preliminary experiments, we found that MLK3 was markedly increased in TAC mice. As it is reported, upregulation of miR-138 plays a protective role in myocardial adaptation to chronic hypoxia by suppressing MLK3 and its downstream targets^30,46^. Xing et al. demonstrated that miR-140-5p aggravates hypoxia-induced injury through upregulation of MLK3^47^. These studies demonstrated that MLK3 can harm cardiomyocytes in vitro, which supports our results.

As a mixed lineage kinase inhibitor, URMC-099 possesses the ability to inhibit the activity of MLK1, MLK2, LRRK2, and MLK3^34,48^, which could act as a useful tool to investigate the role of MLK3 in many other diseases^38–40^. Interestingly, the similar effects of URMC-099 on an established cardiac remodeling were observed in the present study. Inhibition of MLK3, prior to or later to TAC condition, emerged as protective effects on cardiac remodeling, yet the underlying mechanisms are poorly elucidated. Kyoko Tomita et al.^43^ reported the protective effect of URMC-099 on the FFC diet-induced liver fibrosis, which may be caused by reduction of collagen deposition in liver sections. In addition, they also found that URMC-099 could reduce LPS-induced macrophage activation and migration. As well known, small molecule inhibitors can directly reduce the level of phosphorylated protein or decrease the level of total protein, resulting in a decrease in the level of phosphorylated protein^49,50^. However, in most cases, the inhibitors often reduce the phosphorylation as well as total protein. In the present study, although the ratios of p-MLK3/MLK3 and p-JNK/JNK remained unchangeable, their expression of MLK3, p-MLK3, JNK, and p-JNK were significantly decreased by URMC-099, in which the decreased phosphorylated protein that really works exerted the protective function.

However, Calamaras et al.^42^ reported that MLK3 prevented adverse cardiac remodeling in the setting of pressure overload, which partly attributed to MLK3 regulated phosphorylation of the stress-responsive JNK kinase. They also reported that CM-derived MLK3 contributes to the increased MLK3 in HF, basal LV hypertrophy observed in MLK3 depletion mice, although with no changes in cardiac function and structure. The lack of sustained MLK3 elevation after TAC may contribute to pathological remodeling in WT mice and may be taken as an explanation for more advanced remodeling in MLK3 deficiency mice. In this study, we also observed that early increased MLK3 in week 1 and 2 after TAC. Treatment with URMC-099 in vivo not only inhibited the expression of MLK3, but reduced the expression of p-JNK and total-JNK. In conclusion, inhibition of MLK3 could be protective in this condition, which may be partly due to the basic MLK3 and JNK level, different strategies of reducing the expression of MLK3, and samples obtained from time points and observed. However, the mechanism by which MLK3 can affect CHF pathogenesis has not been reported in detail, and at least the contribution of MLK3 in different types of cells is still not clear.

Inflammation is a prominent feature in the early stage of CHF and is marked by increased production of pro-inflammatory cytokines including tumor necrosis factor α, IL-1β, IL-18, MCP-1, MIP1α, CXCL1, and CXCL2, all of which promote inflammation, recruit macrophages, activate fibroblasts, and cause myocardial fibrosis^51,52^. Growing evidence indicates that nonischemic myocardial injury is also associated with inflammation. It has been demonstrated that inflammatory factor production and inflammatory cell infiltration occurs in post-TAC in mice^53,54^. NLRP3 has been reported to participate in the regulation of inflammatory diseases. Once activated, NLRP3 nucleates the assembly of an inflammasome, leading to caspase-1-mediated proteolytic activation of the IL-1β family of cytokines, and inducing an inflammatory form of cell death known as pyroptosis^55^, which promotes myocardial remodeling^56^. MLK3 has been reported to be involved in inflammatory diseases such as psoriasis and in hepatocyte injury^57,58^. NF-κB is activated as an early response to TAC and is known to promote NLRP3 expression^14^. NF-κB is also an important downstream effector of MLK3. Our results show that the expressions of NF-κB and inflammation-related proteins were significantly increased in early TAC, and that this increased expression can be inhibited with URMC-099. In our study, MLK3 knockdown effectively improved cardiac function, and reduced collagen deposition, inflammasome formation, and apoptosis in TAC mice at 7th day. MLK3 silencing can effectively downregulate the expression of NLRP3 in cardiomyocytes of TAC mice at 7th day. And our data showed that MLK3 silencing inhibits NF-κB/NLRP3 signaling pathway-mediated inflammation and pyroptosis. Next, MLK3 silencing reduces macrophage recruitment factor and inflammatory factor levels, including IL-18, MCP-1, MIP1α, CXCL1, and CXCL2. Thus, our data indicate that MLK3 leads to myocardial fibrosis and cardiac dysfunction through NF-κB/NLRP3-mediated inflammation and pyroptosis in the early stage of pressure overload.

Mitochondrial dysfunction and oxidative stress are key factors in the progression of heart failure. Mitochondrial detoxifying systems can counteract excessive accumulation of ROS and MDA to prevent cardiac degeneration upon chronic stress^59,60^. Excessive ROS promotes vascular disease, causes lipid and protein oxidation, causes single stranded DNA breaks, induces cardiac hypertrophic growth, and induces cardiac remodeling and/or ventricular dilatation by dysregulating certain signaling pathways^61,62^. Ferroptosis is a relatively new form of cell death that has been discovered in recent years and occurs in response to the loss of GPX4 activity and subsequent accumulation of lipid-based ROS. These changes cause the mitochondria to become smaller while increasing membrane density. Furthermore, cristae are impaired and there is overall mitochondrial dysfunction^63,64^. Intracellular ROS can be reduced to their corresponding alcohols or water by GPX4-catalyzed GSH. The cystine/glutamate transporter (xCT, also known as SLC7A11) is an important transport carrier of cystine used in GSH synthesis^65,66^. The JNK/cellular tumor antigen p53 is the primary signal effector regulating xCT in oxidative stress. p53 can enhance ferroptosis by inhibiting the expression of xCT^67–69^. JNK also is an important downstream signal for MLK3. Our data show that the expression of JNK and oxidative stress-related proteins, and the extent of ferroptosis were significantly altered by URMC-099 in advanced TAC. Our results show that MLK3 silencing can effectively improve cardiac function, reduce collagen deposition, ROS level, and ferroptosis in TAC mice at 8 weeks and can effectively inhibit the expression of JNK and p53 while increasing GPX4, xCT, and GSH levels. These results indicate that MLK3 may lead to myocardial fibrosis and cardiac dysfunction through JNK/p53-mediated oxidative stress and ferroptosis in the advanced stage of pressure overload.

miRNAs are key regulators of a variety of biological processes and play an important role in regulating mRNA expression^70,71^. We used the Targetscan platform to predict miRNAs that may target MLK3. We chose to focus on miR-351, which has been predicted to have an important role in autophagy, hepatic fibrosis, kidney injury, and microvascular dysfunction^72–75^. However, whether miR-351 plays an important role in CHF has not been reported. We found that, compared with sham mice, miR-351 levels were significantly decreased in TAC mice at all time points, suggesting that miR-351 may be a potential target to modulate MLK3 during CHF. Next, we found a marked decrease in luciferase activity after transfection with miR-351 mimics in cells expressing WT-MLK3, whereas mutation of the MLK3 3′UTR within the miR-351 seed sequence binding site eliminated the response to miR-351. These data indicated that MLK3 is a direct target of miR-351. Lastly, we found that an miR-351 antagomir can effectively improve cardiac function while inhibiting cardiac hypertrophy and fibrosis.

In summary, these results demonstrate miR-351/MLK3 as potential targets to prevent or attenuate cardiac phenotype in pressure overload related falling heart.

## Supplementary information

Supplementary information

Supplementary Figure 1

Supplementary Figure 2

Supplementary Figure 3

Supplementary Figure 4

Supplementary Figure 5

Supplementary Figure 6

Supplementary Figure 7
