# Supplementary material for: Pyroptosis and ferroptosis induced by mixed lineage kinase 3 (MLK3) signaling in cardiomyocytes are essential for myocardial fibrosis in response to pressure overload
Source: Cell Death Dis. 2020 Jul 24;11(7):574. doi: 10.1038/s41419-020-02777-3 (PMC7382480; doi:10.1038/s41419-020-02777-3)
Supplement: Supplementary file 1 — Supplementary information [file 41419_2020_2777_MOESM1_ESM.docx]

**Supplementary information**

**Cell culture and treatment**

HL1 mouse cardiomyoblast cell line was purchased from the Procell Life Science&Technology Co,.Ltd. (Wuhan, China) and cultured in minimum Eagle’s medium (MEM) (Gibco Laboratories, USA) supplemented with 10% fetal bovine serum (FBS) (Gibco Laboratories, USA) and 100 U/ml penicillin/100 mg/ml streptomycin in an atmosphere of 95% air and 5% CO_2_ at 37°C. Medium was replaced every 2 days, and the cells were digested with 0.05% trypsin when the density of the cells reached 80–90%. HL1 cells were seeded in six-well plates or 96-well plates and treated as used for the following experiments.

LPS (Sigma) was dissolved in sterile deionized water and used at a final concentration of 0.5 μg/ml, as an inducer of pyroptosis. The MLK3 inhibitor URMC-099 (MedChemExpress, Shanghai, China) was dissolved in dimethyl sulfoxide (DMSO) and used at a concentration of 200 μM. MCC950 (MedChemExpress, Shanghai, China), as a NLRP3 inhibitor, was dissolved in DMSO and used at a concentration of 50 μM. Fin56 (MedChemExpress, Shanghai, China) was dissolved in DMSO and used at a final concentration of 0.5 μg/ml, as an inducer of ferroptosis, and the ferroptosis inhibitor Ferrostatin-1 (MedChemExpress, Shanghai, China) was used at a concentration of 1μM which dissolved in DMSO.

**Automated western blotting analysis**

Western blotting was performed as SimpleWestern assays using the Wes system using the Size Separation Master Kit with Split Buffer (12–230 kDa) according to the manufacturer’s standard instruction (ProteinSimple), a combination of capillary electrophoresis and immunodetection techniques, and using the Size Separation Master Kit with Split Buffer (12–230 kDa) according to the manufacturer’s standard protocols. Following abtibodies were used, MLK3 (Proteintech, 11996-1-AP), Phospho-NF-κB p65(CST, 4025), NLRP3 (Abcam, ab214185), Cleaved GSDMD (CST, 50928), IL-1 beta (Abcam, ab9722), IL-18 (Abcam, ab71495), Phospho-JNK (Thr183/Tyr185, CST, 9255), p53 (Proteintech, 10442-1-AP), xCT (Proteintech, 26864-1-AP), FTH1 (Proteintech, 10727-1-AP), COX2 (Proteintech, 12375-1-AP) and GPX4 (Proteintech, 14432-1-AP).

**Caspase-1 activity**

Caspase-1 activity was measured in cell lysate, using a Caspase 1 Activity Assay Kit (C1102, Beyotime Biotechnology, Shanghai, China) as described by the manufacturer.

**Supplementary Figure legends**

**Supplementary Figure 1. Inhibition of MLK3 and JNK by URMC-099 and AAV strategy.**

**a** Western blot determination of the expression of mouse MLK3, p-MLK3, JNK and p-JNK in hearts from administration of URMC-099 and injection of AAV^MLK3-^, respectively. GAPDH was used as a control. **b** PVDF membrane image was stained by Ponceau S. **c-h** Relative expression of MLK3, p-MLK3, JNK and p-JNK. Data presented as Mean±SEM, n = 3 biologically independent samples, ***P*< 0.01 vs Isotype control, ##*P* < 0.01 vs AAV^NC^ by Student’s t test.

**Supplementary Figure 2. MLK3 Depletion Reverses Cardiac Dysfunction After 1 week of TAC.**

**a** LV internal dimension at end-diastole (LVID;d). **b** LV internal dimension at end-systole (LVID; s) **c** left ventricular end-diastolic volume (LVEDV). **d** left ventricular end-systolic volume (LVESV) and **e** left ventricular mass (LV mass) of Sham+AVV^NC^, TAC+AVV^NC^ or TAC+AVV^MLK3-^ mice after 1 weeks. n=5 for each group. Data presented as Mean ± SEM, ***P*< 0.01 vs sham-AVV^NC^, ^#^*P* < 0.05, ^##^*P*< 0.01 vs TAC+AVV^NC^ by one-way ANOVA followed by Tukey’s multiple comparisons test.

**Supplementary Figure 3. MLK3 Depletion Reverses Change of inflammation related genes induced by TAC.**

**a** IL-18, **b** IL-1β, **c** MIP1α, **d** MCP-1, **e** CXCL1, **f** CXCL2 and **g** ICAM1 mRNA in whole ventricular lysates as measured by qPCR, normalized for the internal control GAPDH and expressed as fold increase over sham. n=5. Mean±SEM, ***P*< 0.01 vs Sham+AVV^NC^, ^##^*P* < 0.01 vs TAC+AAV^NC^ by one-way ANOVA followed by Tukey’s multiple comparisons test.

**Supplementary Figure 4. MLK3 Depletion Inhibits** **Pyroptosis-Related Protein and mRNAs expression in HL1 cells.**

**a-d** Automated western blotting determination of the expression of mouse MLK3, NLRP3 and GSDMD proteins in HL1 mouse cardiomyoblast cell. GAPDH was used as a control. **e-h** IL-18, IL-1β, ANP and BNP mRNA in HL1 mouse cardiomyoblast cell as measured by qPCR, normalized for the internal control GAPDH and expressed as fold increase over control. **i** Caspase-1 activity in HL1 mouse cardiomyoblast cell. Mean±SEM, n = 3, ***P*< 0.01 vs control, ^##^*P* < 0.01 or ^#^*P* < 0.05 vs LPS intervention by one-way ANOVA followed by Tukey’s multiple comparisons test.

**Supplementary Figure 5. MLK3 Depletion Reverses Cardiac Dysfunction After 8 weeks of TAC.**

**a-e** LV internal dimension at end-diastole (LVID;d), LV internal dimension at end-systole (LVID;s), left ventricular end-diastolic volume (LVEDV), left ventricular end-systolic volume (LVESV) and left ventricular mass (LV mass) of Sham+AVV^NC^, TAC+AVV^NC^ or TAC+AVV^MLK3-^ mice after 8 weeks. n=5 for each group. Mean ± SEM, fibrotic area control values were set to 1. ***P*< 0.01 vs sham-AVV^NC^, ^#^*P* < 0.05, ^##^*P*< 0.01 vs TAC+AVV^NC^ by one-way ANOVA followed by Tukey’s multiple comparisons test. **f-g** MDA, T-SOD and GSH in whole ventricular lysates as measured by colorimetric method, n=5. Mean±SEM, ***P*< 0.01 vs Sham+AVV^NC^, ^##^*P* < 0.01 vs TAC+AAV^NC^ by one-way ANOVA followed by Tukey’s multiple comparisons test.

**Supplementary Figure 6. MLK3 Depletion Inhibits Ferroptosis- and Oxidative Stress-Related Protein Expression in HL1 cells.**

**a-f.** Automated western blotting determination of the expression of mouse MLK3, p-JNK, p53 COX2 and FTH1 proteins in HL1 mouse cardiomyoblast cell. GAPDH was used as a control. **g-i** MDA, T-SOD and GSH levels in HL1 mouse cardiomyoblast cell as measured by colorimetric method. **j-k** ANP and BNP mRNA in HL1 mouse cardiomyoblast cell as measured by qPCR, normalized for the internal control GAPDH and expressed as fold increase over control. Mean±SEM, n = 3, ***P*< 0.01 vs control, ^##^*P* < 0.01 or ^#^*P* < 0.05 vs FIN56 intervention by one-way ANOVA followed by Tukey’s multiple comparisons test.

**Supplementary Figure 7. miR-351 Inhibits MLK3 Expression to Improve Cardiac Function.**

**a-e** LV internal dimension at end-diastole (LVID;d), LV internal dimension at end-systole (LVID;s), left ventricular end-diastolic volume (LVEDV), left ventricular end-systolic volume (LVESV) and left ventricular mass (LV mass) of Sham+AVV^NC^, TAC+AVV^NC^ or TAC+AVV^MLK3-^ mice after 8 weeks. n=5 for each group. Mean ± SEM, fibrotic area control values were set to 1. ***P*< 0.01 vs Sham, ^#^*P* < 0.05, ^##^*P*< 0.01 vs TAC by one-way ANOVA followed by Tukey’s multiple comparisons test. **f-i** ANP, BNP, MMP2, and MMP9 mRNAs in whole ventricular lysates as measured by qPCR, normalized for the internal control GAPDH and expressed as fold increase over Sham+AVV^NC^. n=5. Mean±SEM, ***P*< 0.01 vs Sham, ^##^*P* < 0.01 vs TAC by one-way ANOVA followed by Tukey’s multiple comparisons test.
