# Supplementary figures and images for: Pyroptosis and ferroptosis induced by mixed lineage kinase 3 (MLK3) signaling in cardiomyocytes are essential for myocardial fibrosis in response to pressure overload
Source: Cell Death Dis. 2020 Jul 24;11(7):574. doi: 10.1038/s41419-020-02777-3 (PMC7382480; doi:10.1038/s41419-020-02777-3)

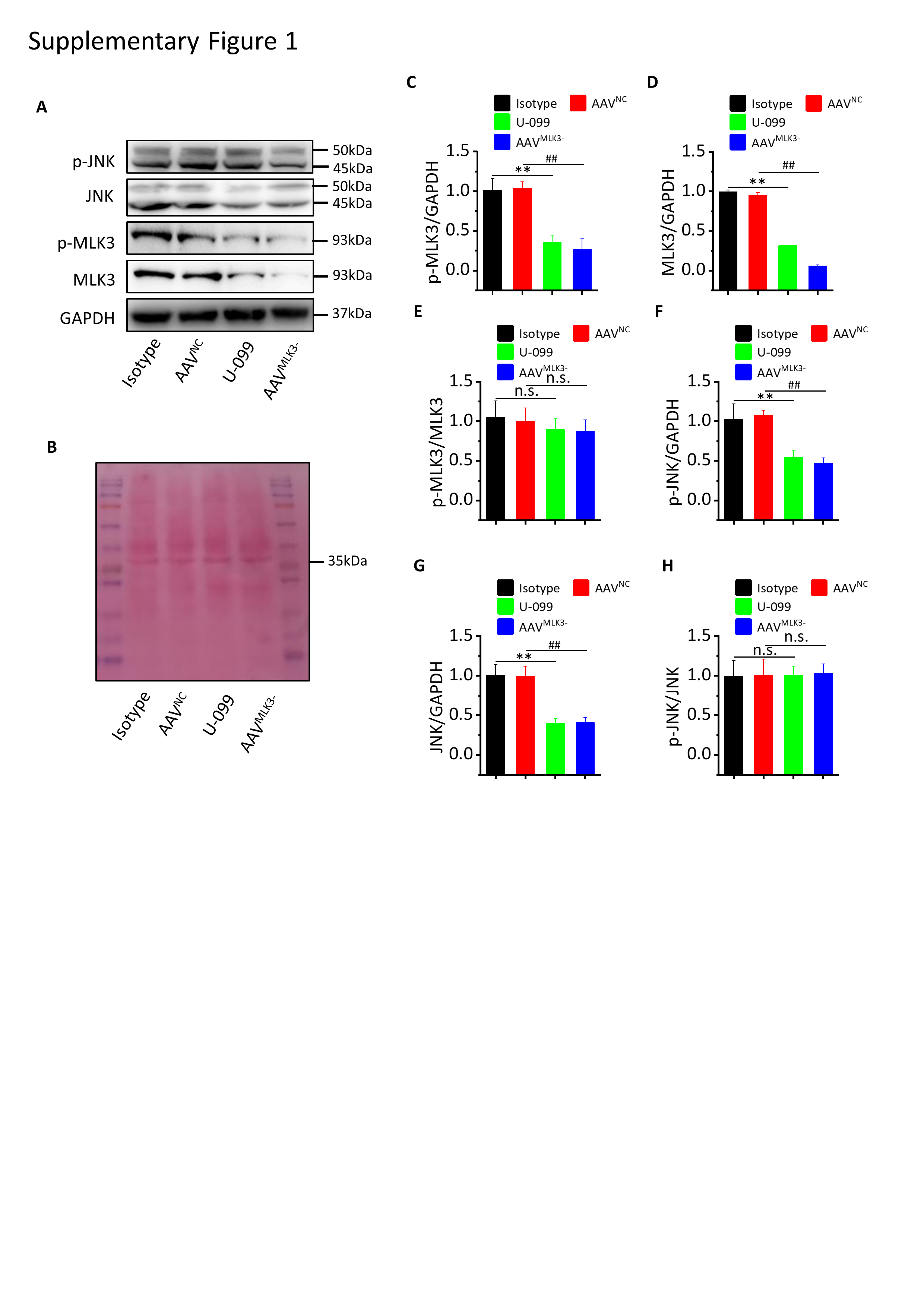

Supplement: Supplementary file 2 — Supplementary Figure 1 [file 41419_2020_2777_MOESM2_ESM.tif]

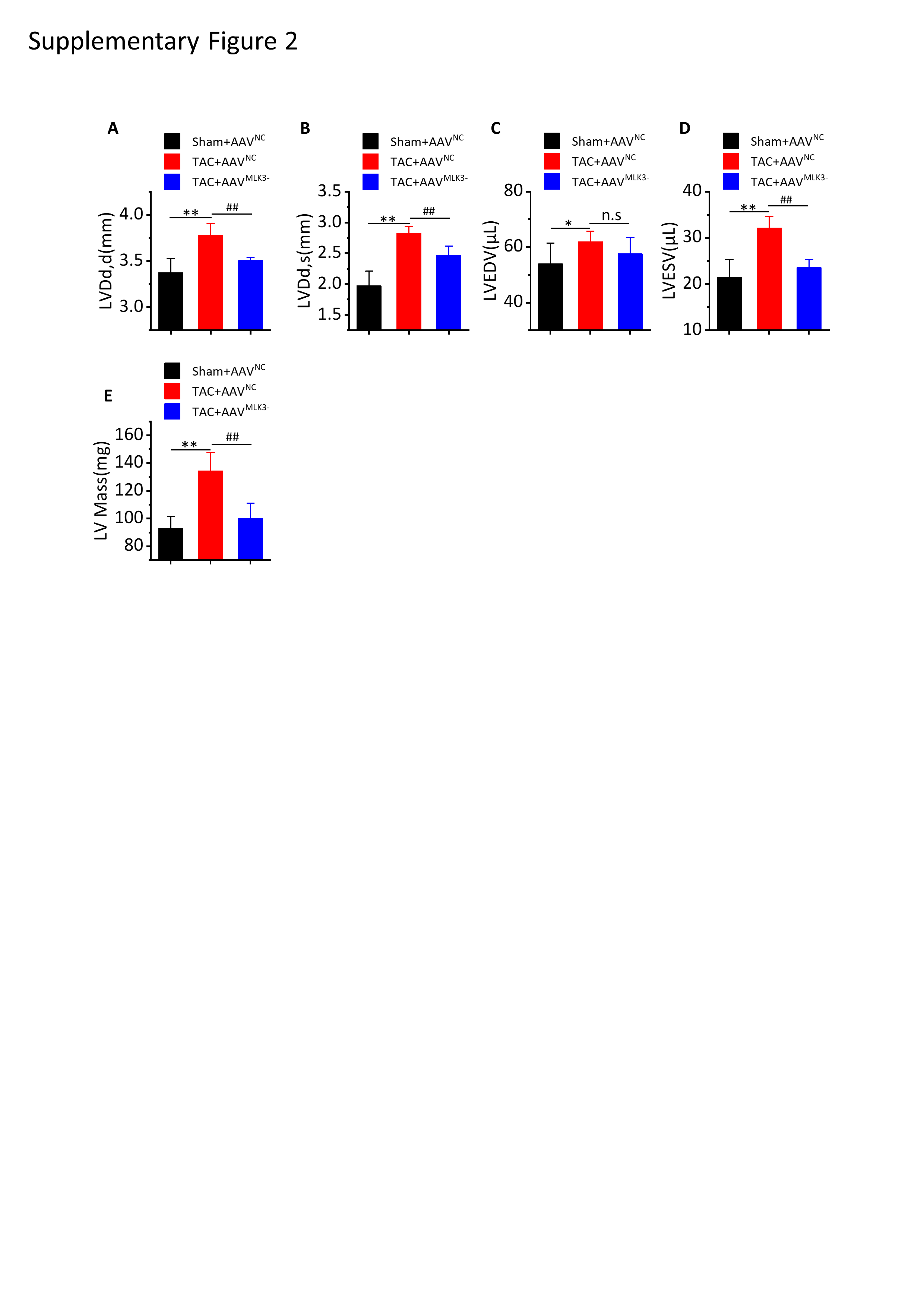

Supplement: Supplementary file 3 — Supplementary Figure 2 [file 41419_2020_2777_MOESM3_ESM.tif]

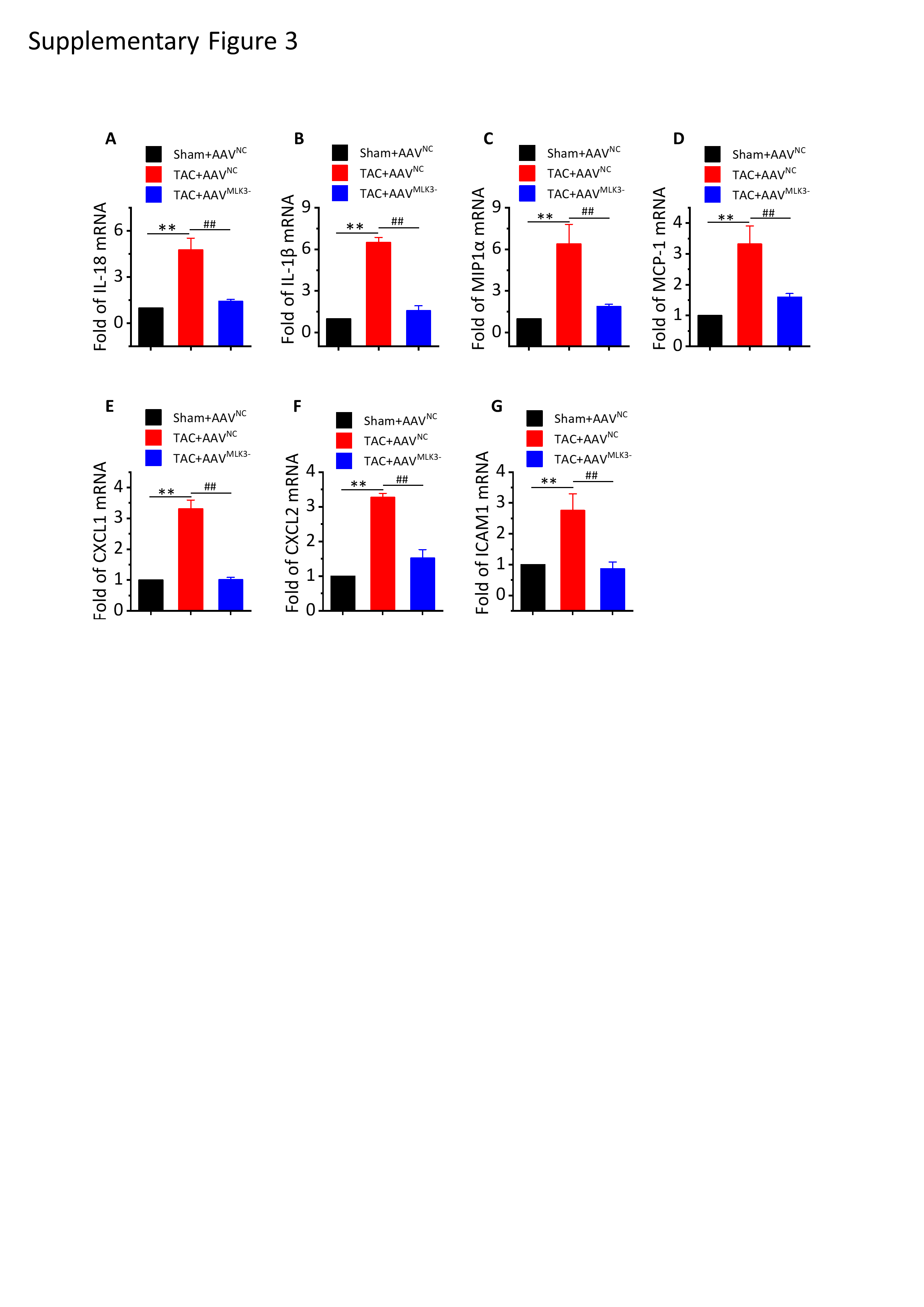

Supplement: Supplementary file 4 — Supplementary Figure 3 [file 41419_2020_2777_MOESM4_ESM.tif]

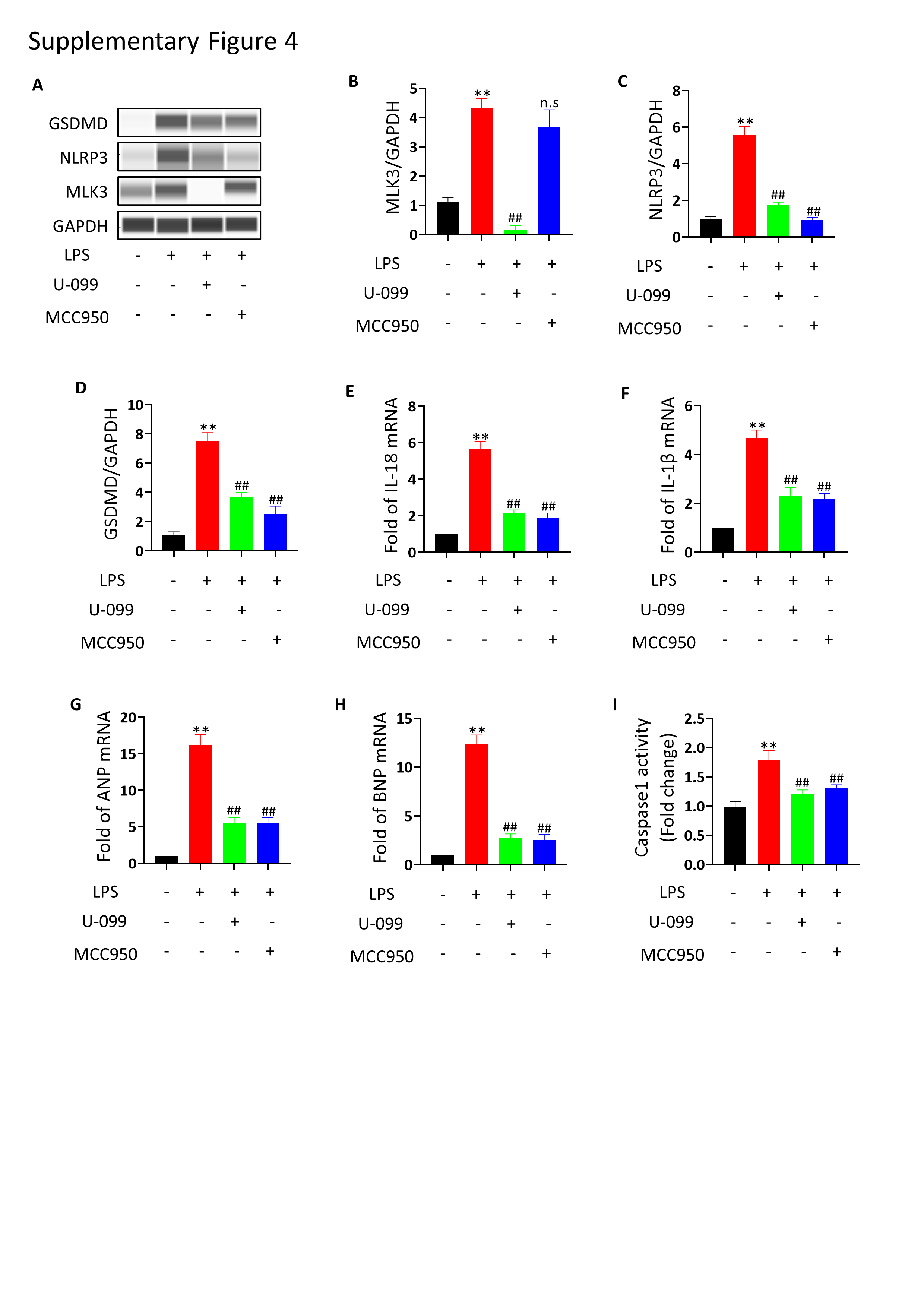

Supplement: Supplementary file 5 — Supplementary Figure 4 [file 41419_2020_2777_MOESM5_ESM.tif]

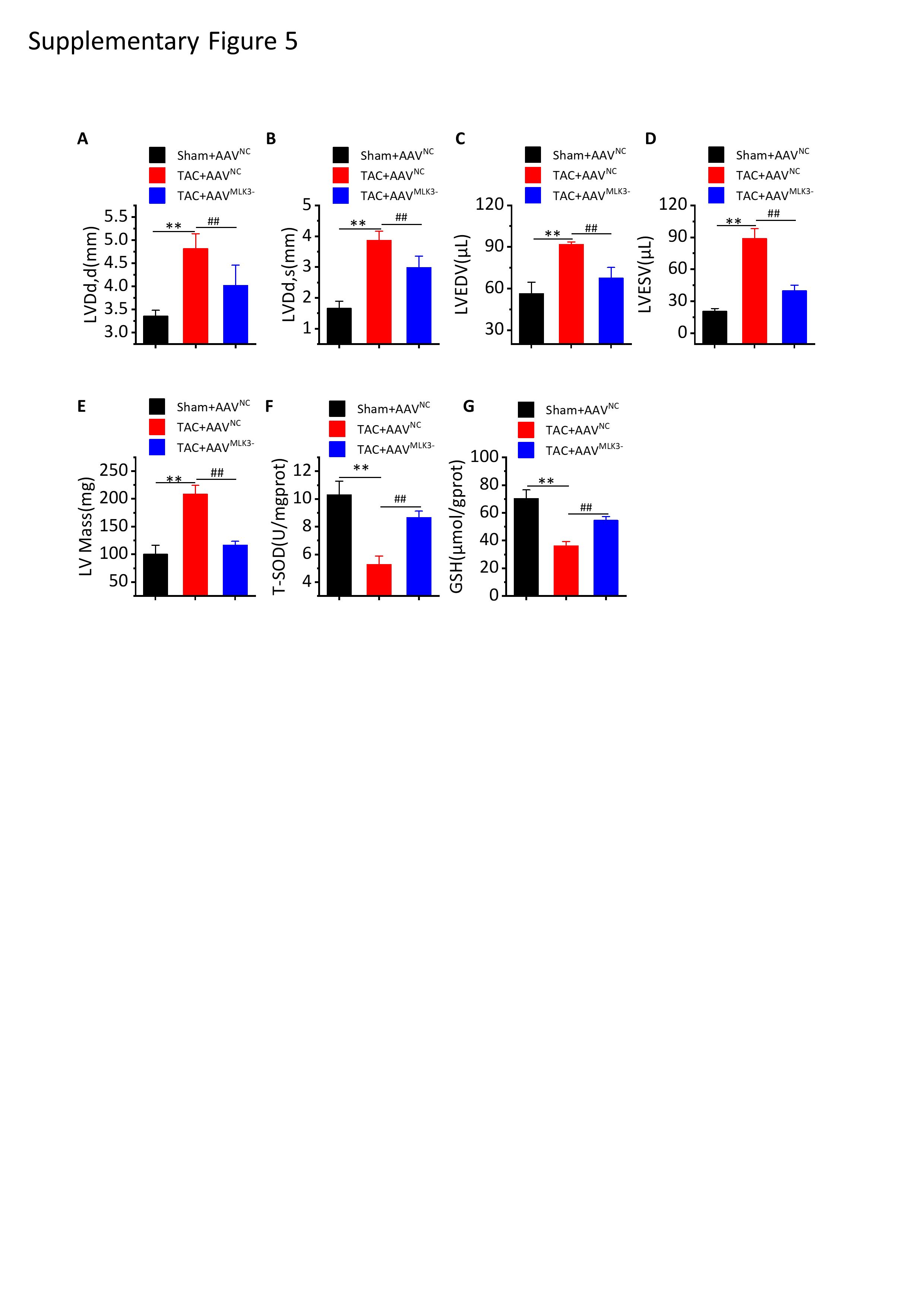

Supplement: Supplementary file 6 — Supplementary Figure 5 [file 41419_2020_2777_MOESM6_ESM.tif]

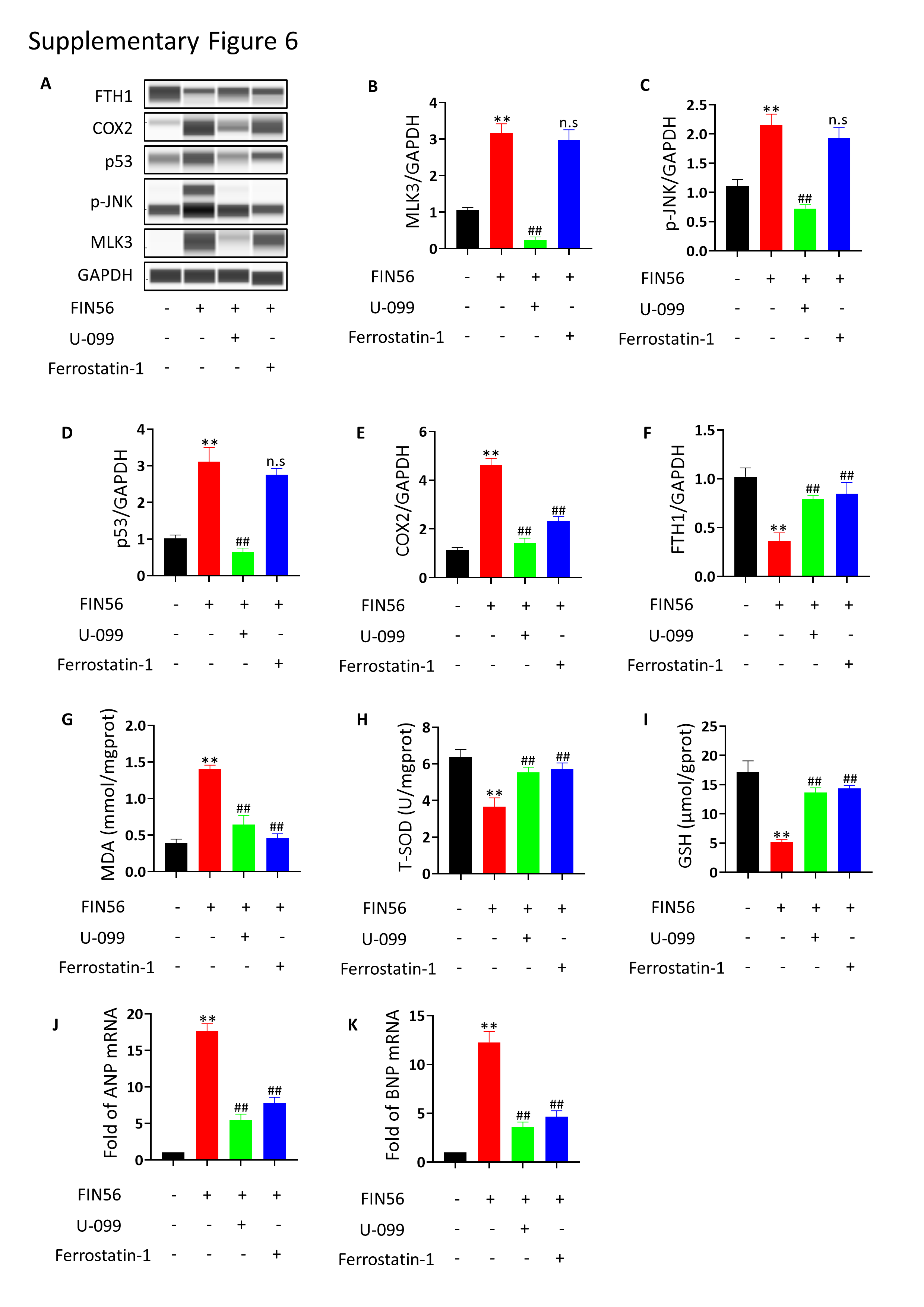

Supplement: Supplementary file 7 — Supplementary Figure 6 [file 41419_2020_2777_MOESM7_ESM.tif]

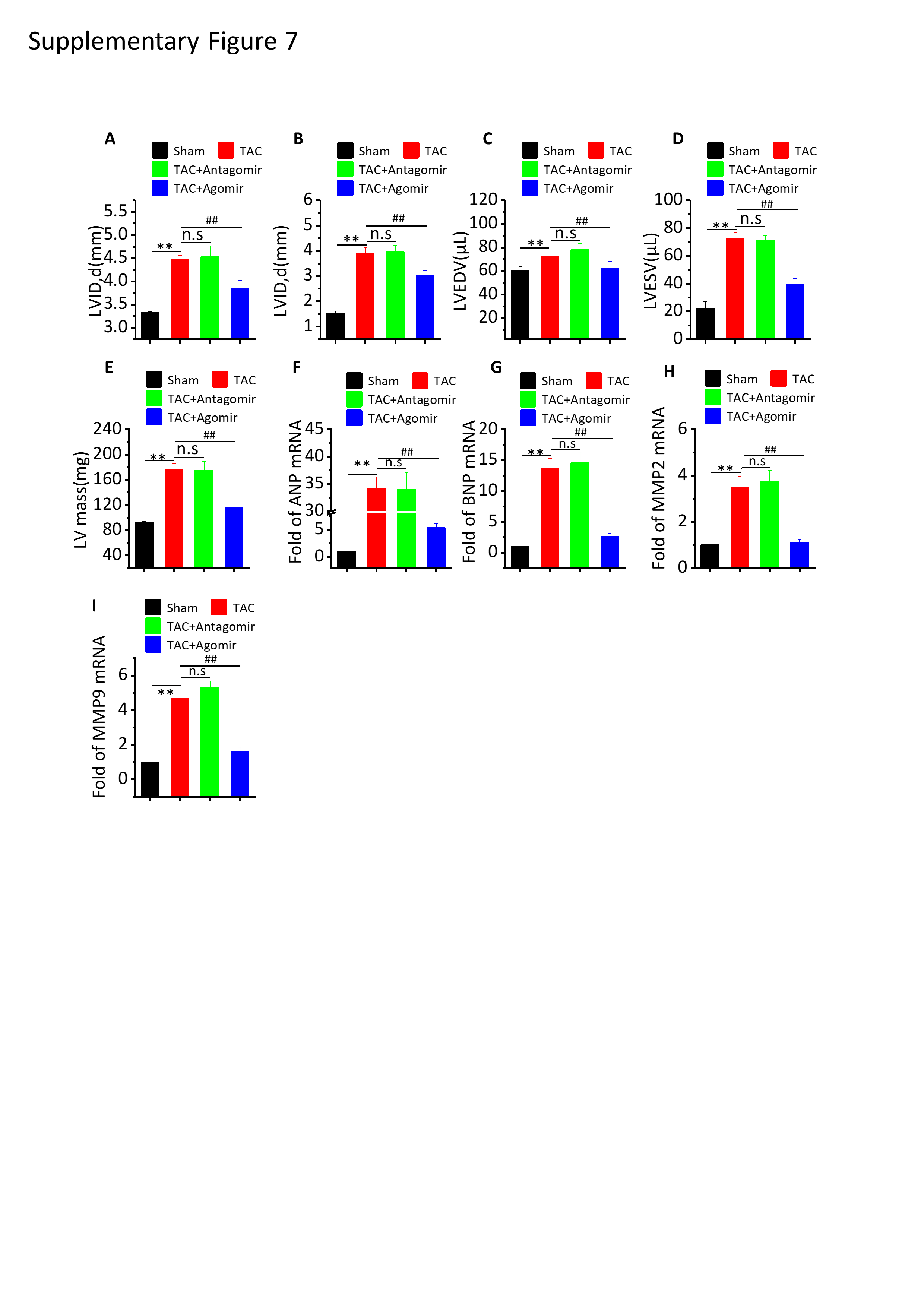

Supplement: Supplementary file 8 — Supplementary Figure 7 [file 41419_2020_2777_MOESM8_ESM.tif]
